# Machine learning-based gait classification and genome-wide association identify a QTL for gait type in Colombian paso horses

**DOI:** 10.1016/j.isci.2026.116320

**Published:** 2026-06-11

**Authors:** Miguel Novoa-Bravo, Jennifer R.S. Meadows, Filipe Serra-Bragança, Britt van de Vall, Klas Kullander, Marie Rhodin, Gabriella Lindgren

**Affiliations:** 1 Genética Animal de Colombia SAS, Bogotá, Colombia; 2 Department of Animal Biosciences, Swedish University of Agricultural Sciences, P.O. Box 7023, 75007 Uppsala, Sweden; 3 Department of Medical Biochemistry and Microbiology, Uppsala University, 75123 Uppsala, Sweden; 4 SciLifeLab, Uppsala University, 75123 Uppsala, Sweden; 5 Department of Clinical Sciences, Faculty of Veterinary Medicine, Utrecht University, Utrecht 3584CM, the Netherlands; 6 Department of Immunology, Genetics and Pathology, Uppsala University, Uppsala, Sweden

**Keywords:** genetics, developmental neuroscience, machine learning

## Abstract

Coordinated mammalian locomotion relies on spinal circuits where *DMRT3* regulates strides and alternative gaits. However, *DMRT3* does not explain differences between the Colombian paso horse breed’s specialized gaits, Colombian trocha and Colombian trot. We used inertial sensors and machine learning to develop accurate phenotyping (*n* = 225 horses), before performing genome-wide association analysis (*n* = 85 horses, 670K array). We identified a 2.43 Mb quantitative trait locus (QTL) on ECA16, where haplotypes featuring lead variants (rs1147402472, *p* = 1.95 × 10^−8^; rs1136628503, *p* = 8.52 × 10^−8^) explained 48.6% of gait variance (*p* < 0.001). The QTL contained 11 genes linked to neurodevelopment and muscle regulation, including *LHFPL4, SRGAP3,* and *ATP2B2*. Interaction analyses suggested a functional link between the latter genes. rs1147402472-C was common across diverse gaited horse breeds, but the Colombian trot-specific haplotype (rs1147402472-C/rs1136628503-T) was absent elsewhere. These findings demonstrate that fine-scale genetic differentiation at ECA16 underlies neural adaptations distinguishing complex locomotor traits and highlight the power of AI-assisted phenotyping in genomics.

## Highlights

• AI-LSTM reclassified 50% of gaits, surpassing subjective breeder classification
• A 2.43 Mb QTL on ECA16 distinguishes the Colombian trocha from Colombian trot gait
• This QTL regulates locomotion independently of the known *DMRT3* mutation
• Interaction of *ATP2B2* and *SRGAP3* likely regulates locomotion in these horses

## Graphical abstract

**Figure:**
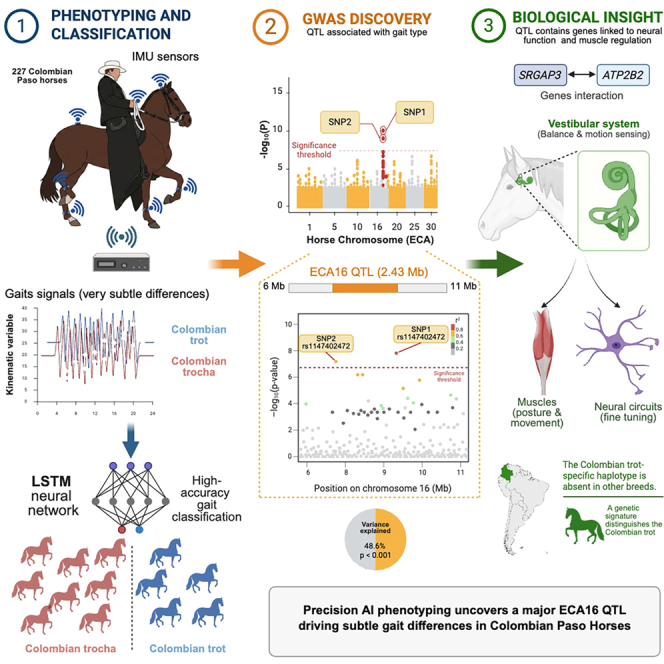

## Introduction

The study of gait genetics in mammals offers valuable insights across two complementary domains: understanding human locomotor disorders and exploring the evolutionary innovations that underpin efficient movement.^1^^,^^2^ In humans, conditions such as cerebral palsy, muscular dystrophy, and spinal cord injuries reveal how genetic and neurological factors govern coordinated motion and how their disruption can lead to debilitating outcomes.^1^ Comparative studies in non-human mammals provide a dual benefit. On one hand, they offer models for studying fundamental locomotor mechanisms that may parallel human pathology. On the other hand, they illuminate the evolutionary trajectories through which species have developed specialized gaits to maximize efficiency and adaptability. For example, elephants use a four-beat ambling gait that minimizes energy expenditure by evenly distributing weight,^3^ while giraffes rely on a completely lateral two-beat gait and achieve speed primarily through increased stride length rather than frequency.^4^ Such strategies, shaped by natural selection, highlight the biomechanical constraints and adaptations that influence gait across species, offering both biomedical relevance and evolutionary insight.

Horses provide an exceptional model for studying the genetics of locomotion due to their well-documented breed-specific gait variations, moderate-to-high heritability of locomotion traits (e.g., h^2^ for movement quality and gait symmetry, French saddle breeds: 0.10–0.69^5^; Belgian Warmbloods: 0.33–0.52^6^), and the availability of high-resolution genomic tools.^7^ Most horses perform three natural gaits: walk, trot, and canter. Furthermore, certain breeds, collectively known as gaited horses, exhibit additional smoother gaits and have been selectively bred for rider comfort and endurance.^8^ These gaits include lateral (tölt, pace, marcha picada, and paso fino) or diagonal coupled gaits (foxtrot, marcha batida, Colombian trocha, and Colombian trot). Like natural gaits, it is possible for a single horse to perform multiple additional gaits, e.g., *tölt* and *pace* in Icelandic horses, but the genetics underlying these abilities is complex.^9^

The discovery of a nonsense mutation in *DMRT3* (NM_001317265.1:g.22999655C>A, rs1150690013) marked a breakthrough in equine locomotion genetics, demonstrating that this mutation enables lateral ambling gaits.^10^^,^^11^^,^^12^ Horses carrying the nonsense *DMRT3* variant show altered spinal cord neuron development, which affects stride patterns and enhances coordination in lateral footfall sequences.^10^ While *DMRT3* is a key determinant of lateral gait ability in several breeds like Colombian paso fino (paso fino), Peruvian paso (paso llano), Icelandic horses (tölt and pace), the Mangalarga Marchador (*marcha picada*), and others,^11^ this locus does not explain all horse gaits.

Several breeds perform a distinct diagonal coupled gait known as the “broken trot,”^9^ characterized by a slight negative diagonal advance placement, meaning the forelimb contacts the ground just before the contralateral hindlimb. This gait is observed in breeds such as the Karbarda, Transbaikal and Yakut (all tropota gaited), Akhal-Teke (glide gait), and Marwari (revaal gait), and it is not associated with *DMRT3*.^13^^,^^14^^,^^15^ Additional broken trot gaits can be observed, for example, the Brazilian Mangalarga Marchador and Campolina horses perform marcha batida, a diagonal ambling gait,^16^ while the Colombian trocha horse exhibits Colombian trocha gait.^17^ The lack of *DMRT3* association with gait in all these breeds suggests that alternative genetic pathways contribute to their gait mechanics, underscoring a gap in our understanding of equine locomotion genetics. To date, no common genetic mechanism has been identified across the various diagonal coupled gaits. In Colombian paso horses (CPHs), these diagonal gaits are often observed within the same lineages, suggesting a high degree of co-inheritance and a shared polygenic architecture that is independent of the *DMRT3* locus.

CPHs presents a unique opportunity to explore the genetic basis of gait diversity. This breed has been shaped by natural and artificial selection to navigate Colombia’s challenging topography, favoring smooth and energy-efficient gaits for long-distance travel. The Colombian Federation of Horse Associations (Fedequinas) has been managing the breed since 1946, with over 277,000 registered horses today (https://www.fedequinas.org; https://www.fao.org/dad-is/). CPHs are classified into four breeds—Colombian paso fino horses (CPF), Colombian trocha horses (CTR), Colombian trocha and gallop horses (CTRG), and Colombian trot and gallop horses (CTG)—based on gait and conformation traits.^17^ There are restrictions and overlaps in the gaited abilities of the four breeds. The ambling *paso fino* gait is breed restricted (to CPF), as is the Colombian trot gait (to CTG). Colombian trot gait consists of an isochronal two-beat and diagonally coupled gait, short strides, and high stride frequency, and there are either two limbs or four limbs in stance phase.^17^ In contrast, two breeds (CTR and CTRG) can perform the Colombian trocha gait, a lateral sequence four-beat and diagonally coupled gait, in which the forelimb hit the ground before the contra lateral hindlimb, and it has a non-isochronal beat pattern; a separate breed pairing (CTG and CTRG) can perform Colombian canter, a gait with shorter strides and vertical sacrum displacement than a regular canter.^17^

Despite the phenotypic complexity of these gaits, classification in CPHs has historically relied on subjective evaluation. Breeders and judges use auditory and visual assessments, listening to the rhythm of footfalls on wooden boards and analyzing movement traits to distinguish between gaits. However, these evaluations are influenced by environmental factors and human perception biases. The rapid stride frequency of CPHs (2.08–2.97 strides per second) further complicates manual classification, making it difficult to discern subtle distinctions between gaits.^17^ This poses a challenge for breeding programs, as reliable gait classification is essential for genetic studies and ensuring horses are trained according to their natural gait predispositions.

To address the real-time subjective nature of gait classification, we used slow-motion video capture and objective kinematic analyses to develop a machine learning-driven gait classification. This machine learning phenotype was then combined with high-density genomic data to investigate the genetic basis of gait differentiation in CPHs. By employing genome-wide association studies (GWASs) alongside advanced locomotion analysis, we identified a major quantitative trait locus (QTL) associated with gait type that operates independently of *DMRT3*. These findings provide insights into the genetic architecture of coordinated movement, shedding light on traits historically selected in gaited horses and offering broader implications for mammalian locomotion.

## Results

### Objective phenotyping and machine learning reclassified the gait type performed by CPHs

**Figure 1:**
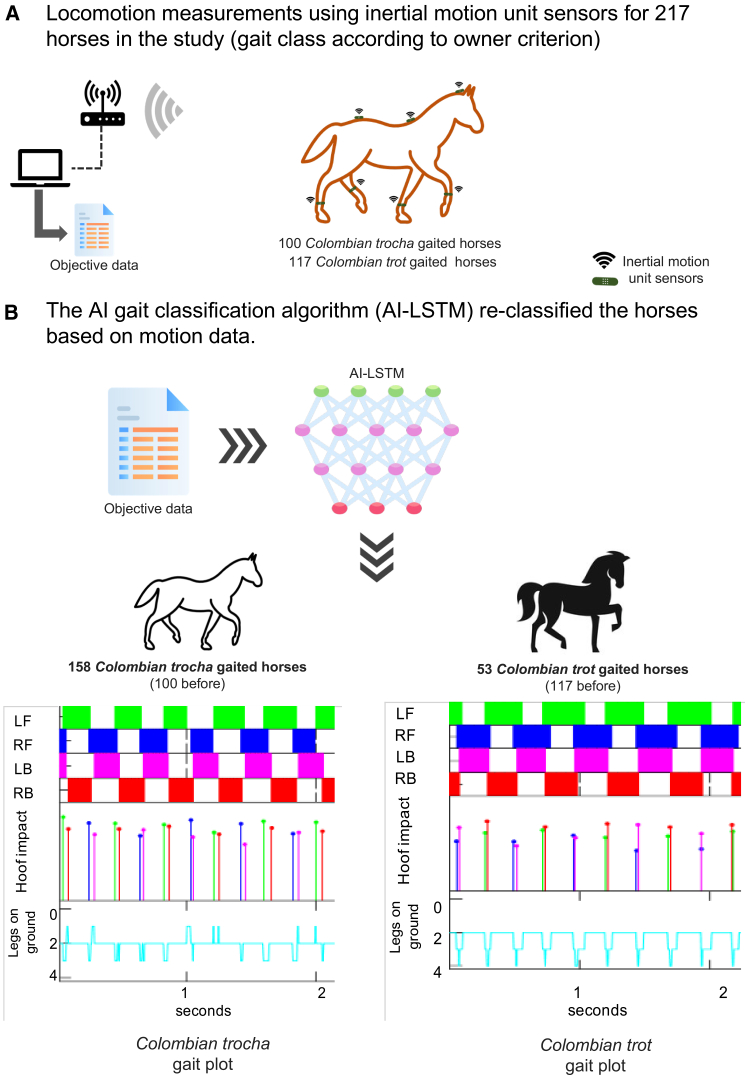
Data collection and gait classification (Colombian trocha or Colombian trot) for 217 Colombian paso horses (A) Locomotion of 217 horses was measured using motion sensors; gait type per horse according to breeder criterion. (B) Gait classification is based on the long-short term memory (AI-LSTM) neural network algorithm, using the 34 locomotion variables analyzed on the same 217 horses and the main differences between groups in footfall plotting. “Hoof impact” is the moment when the hoofs just hit the ground. “Legs on the ground” is the number of limbs that are on the ground at the same time. Footfall timings. Colored, stances; white, swings. LF, left front limb (green boxes); RF, right front limb (blue boxes); LB, left hindlimb (pink boxes); RB, right hindlimb (red boxes). One stride is the 4 limbs in sequence.

We captured 226,868 strides using inertial measurement units (IMUs) from 225 horses (Table 1; Figure 1A). After data quality control (QC) and selecting locomotion segments, we isolated 7,427 strides (averaging 34 per horse) for analysis, focusing on Colombian trocha and Colombian trot gaits. Key differences between Colombian trocha and Colombian trot gaits are highlighted in the footfall plots (Figure 1B). In Colombian trocha gait, the front limb contacts the ground before the contralateral hindlimb (see hoof impact in Figure 1B), which is in contrast to the Colombian trot gait*,* where diagonal hoof impact is almost at the same time (diagonal advance placement close to 0). The Colombian trot gait quadrupedal stance phase also distinguishes this gait from *Colombian trocha* (see legs on the ground in Figure 1B).

**Table 1:** Number of Colombian paso horses selected in this study performing a particular gait per sex

Gait	Females	Males	Total
*Colombian trot*	64	53	117
*Colombian trocha*	55	45	100
*Paso fino*	6	2	8
Grand total	125	100	225

The artificial intelligence (AI)-machine learning long-short term memory (AI-LSTM) neural network algorithm resulted in the reclassification of approximately 50% of horses previously subjectively categorized by breeders. AI-LSTM classified 45% fewer horses as performing Colombian trot gait and 58% more horses as performing Colombian trocha gait (Figure 1B). Six horses were misclassified using the AI-LSTM method. In addition, significative mean differences were identified in 30 of 34 locomotion variables when contrasting AI-LSTM-classified Colombian trocha and Colombian trot gaits (Table S2). The main differences between gaits were sustained in the stance duration (SD)/duty factor (DF), stride duration (STRD), quadrupedal stance (QS), and diagonal advance displacement (DAP). Notably, the use of inertial sensors enabled the detection of subtle variations in DAP, QS, or other variables that exceed the resolution of human perception. While human visual temporal resolution is generally limited to differences above 6–10 ms in laboratory conditions and 20–30 ms in non-laboratory settings,^18^ perhaps as traditional horses show, the measured DAP differences were approximately 13 ms for Colombian trot and 17 ms for Colombian trocha, while QS was as low as 6 ms in Colombian trot. These results suggest that subjective misclassification occurs because these critical kinematic parameters fall at or below the threshold of human sensory detection.

Principal-component analysis (PCA) was used to reduce the dimensionality of the 34 locomotion variables, with 64% of variance captured by principal components (PCs) 1, 2, and 3 (Figure S1). The remaining variability (36%) is due to other factors not captured by the main components identified. Key variables with high contribution to the variance included stride frequency, stance phase per limb, and the number of limbs in contact with the ground. Together, these findings demonstrate biomechanical distinctions between Colombian trocha and Colombian trot, providing quantitative evidence to support their classification.

### A QTL associated with gait type (Colombian trocha*/*Colombian trot) in CPHs

**Figure 2:**
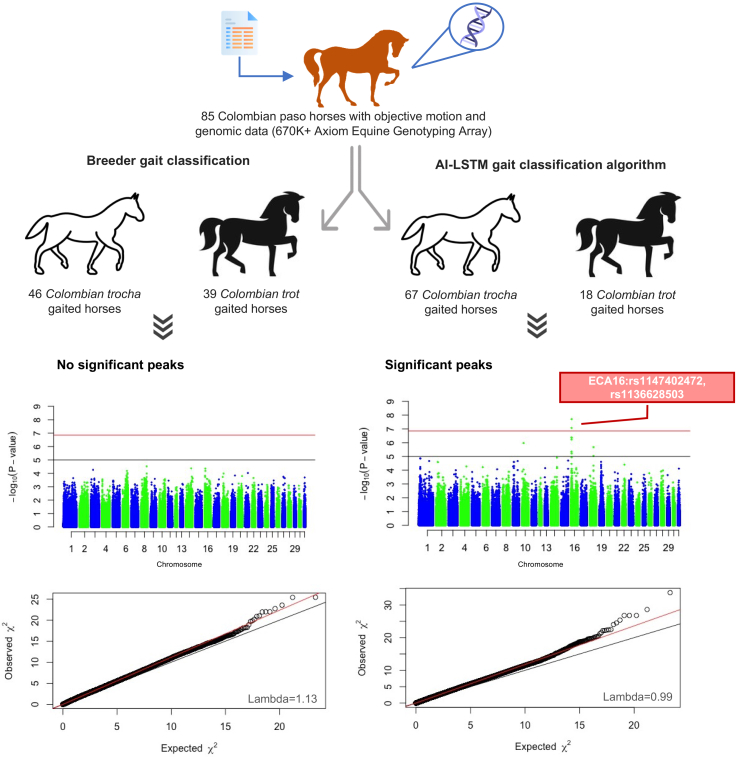
Genome-wide analysis for gait type (Colombian trocha*/*Colombian tro*t*) on 85 Colombian paso horses and their Manhattan plots Two groups of horses were contrasted based on the breeders’ classification and the AI-LSTM neural network classification. Two lead SNPs (SNP1: rs1147402472 and SNP2: rs1136628503) were found only with the AI-LSTM classification group (red box). A polygenic_hglm mixed model-structured association approach was used. Black line, suggestive threshold (*p* < 1.0 × 10^−5^); red line, significant Bonferroni threshold (*p* < 1.3 × 10^−7^).

For the analysis of gait, we wanted to exclude the potential confounder of the known *DMRT3* locomotive-associated variant, rs1150690013*-*A*.* From a starting set of 217 CPHs, 194 were homozygous rs1150690013-C, 21 horses were carriers of the rs1150690013-A variant, and 2 horses were not genotyped (Table S3). This carrier frequency is consistent with previous reports.^17^ Ninety-five homozygous rs1150690013-C horses were genotyped using the 670K array, and after QC, 360,755 SNPs and 85 horses remained for association analyses (Figure 2). A PCA based on genomic data showed the presence of subtle genetic differences between the two groups of AI-LSTM classified horses (Figure S2; PC1 = 3.5%).

**Figure 3:**
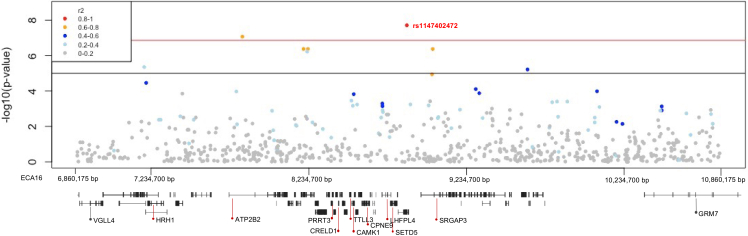
Zoom-in of the associated region on ECA16 of the genome-wide analysis for gait type (Colombian trocha*/*Colombian trot) on 85 Colombian paso horses Known (black) and trait candidate genes (red) are illustrated, and GWAS results are colored for LD (*r*^2^) to the SNP1 (rs1147402472). GWAS significance thresholds are given. Bonferroni significance: red, *p* < 1.3 × 10^−7^; suggestive significance: black, *p* < 1.0 × 10^−5^.

No significant results were found when breeder-classified gaits were used as the phenotype in a polygenic mixed model GWAS. However, two genome-wide significant (*p* < 1.0 × 10^−8^) and eleven suggestive (*p* < 1.0 × 10^−5^) variants were identified when the AI-LSTM objective classification phenotype was used (Figure 2; Table 2). Focusing on the major ECA16 QTL (Figure 3), the lead variant SNP1, rs1147402472, remained consistently significant across multiple GWAS models (Table S4). SNP1 is in an intergenic region between *THUMPD3* and *SRGAP3* (SNP1: ECA16:8,860,175; *p* = 1.95 × 10^−8^). Being the next most significant variant, SNP2 (rs1136628503) lies within intron 1 of *ATP2B2* (ENSECAT00000037909.2) (SNP2: ECA16:7,820,050; *p* = 8.52 × 10^−8^). Frequencies of the variants per gait are shown in Table 3. A larger 2.43 Mb QTL region was defined based on pairwise linkage disequilibrium (LD) to SNP1 and GWAS significance (*r*^*2*^ ≥ 0.6 and *p* value < 1.0 × 10^−5^; Table S6; Figure 3). This region encompassed a total of 32 genes, where *SRGAP3*, *ATP2B2*, and additional nine genes were expressed in brain tissue, including in neuronal and astrocyte cell types (Tables 4 and S5).

**Table 2:** GWAS summary of polygenic mixed model GWAS, binary AI-LSTM phenotype. *p* threshold = 1.0 × 10^-5^

SNP	*p*	Chr:bp	ref/alt	Associated allele	Effect (BETA (SE))
rs1147402472 (SNP1)	1.95 × 10^−8^	16:8860175	T/C	C	−0.55 (0.09)
rs1136628503 (SNP2)	8.52 × 10^−8^	16:7820050	C/T	T	−0.63 (0.11)
rs1141932866	4.21 × 10^−7^	16:8206836	G/A	G	−0.48 (0.09)
rs1145581384	4.21 × 10^−7^	16:8234676	C/T	T	−0.48 (0.09)
rs1151964115	4.26 × 10^−7^	16:9024349	T/C	C	−0.56 (0.11)
rs394798521	5.94 × 10^−7^	16:8229667	G/A	A	−0.40 (0.08)
rs68943671	1.07 x 10-^6^	10:18661325	G/A	A	−0.44 (0.09)
rs1144800763	2.11 x 10-^6^	18:45578012	A/C	C	−0.49 (0.10)
rs396587640	4.45 × 10^−6^	16:7196846	G/A	A	−0.56 (0.12)
rs1140383912	6.05 × 10^−6^	16:9624512	C/T	T	−0.55 (0.12)
rs396793867	1.14 × 10^−5^	16:9020017	A/G	G	−0.41 (0.09)

**Table 3:** Frequencies of the significant GWAS variants SNP1 (rs1147402472) and SNP2 (rs1136628503) per gait based on AI_LSTM classification (Colombian trocha, *n* = 67; Colombian trot, *n* = 17)

Gait	SNP1-C	SNP1-T	SNP2-C	SNP2-T
*Colombian trot*	0.44	0.56	0.67	0.32
*Colombian trocha*	0.06	0.94	0.97	0.03

**Table 4:** Genes within the ECA16 QTL for gait type and their potential function in the brain

Gene	ID	Chr:bp	Human Protein Atlas - brain expression cluster (RNA)	Human Protein Atlas – single-cell type specificity
Histamine receptor H1 (*HRH1*)	ENSECAG00000004544	16:7283054–7364165	neurons - mixed function (mainly)	secretory cells, granulosa cells, glandular and luminal cells, excitatory neurons, astrocytes
ATPase plasma membrane Ca^2+^ transporting 2 (*ATP2B2*)	ENSECAG00000023528	16:7772331–8123695	neurons & synapses - synaptic function (mainly)	excitatory neurons, inhibitory neurons, astrocytes, horizontal cells, bipolar cells, oligodendrocyte precursor cells
Proline rich transmembrane protein 3 (*PRRT3*)	ENSECAG00000014610	16:8385829–8396042	neurons - mixed function (mainly)	rod photoreceptor cells, horizontal cells
Protein disulfide-isomerase (*CRELD1*)	ENSECAG00000014826	16:8393339–8401391	neurons - synaptic function (mainly)	astrocytes, excitatory neurons, inhibitory neurons, oligodendrocytes
Actin-related protein 2/3 complex subunit 4 (*TTLL3*)	ENSECAG00000003474	16:8465465–8484263	brainstem - mixed function (mainly)	low cell type specificity
ADA3 homolog (*TADA3*)	ENSECAG00000018186	16:8497607–8507370	astrocytes - mixed function (mainly)	low cell type specificity
Calcium/calmodulin dependent protein kinase I (*CAMK1*)	ENSECAG00000023628	16:8510815–8527953	neurons - mixed function (mainly)	monocytes, Hofbauer cells
Copine family member 9 (*CPNE9*)	ENSECAG00000007471	16:8552350–8571912	hindbrain - mixed function (mainly)	late spermatids, excitatory neurons
LHFPL tetraspan subfamily member 4 (*LHFPL4*)	ENSECAG00000038489	16:8693050–8747375	neurons - mixed function (mainly)	inhibitory neurons, excitatory neurons, bipolar cells, oligodendrocyte precursor cells, horizontal cells, rod photoreceptor cells
SET domain containing 5 (*SETD5*)	ENSECAG00000016843	16:8744458–8787229	non-specific - transcription (mainly)	oligodendrocyte precursor cells
SLIT-ROBO Rho GTPase-activating protein 3 (*SRGAP3*)	ENSECAG00000010118	16:8927898–9169815	non-specific - transcription (mainly)	oligodendrocyte precursor cells

To address if these GWAS results were impacted by a case-control imbalance and the binary interpretation of the AI-LSTM objective classification, a SAIGE^19^ analysis was performed. Here, the phenotypes were the average predicted probability for each gait per horse (Colombian trocha or Colombian trot), which were derived from the neural network classification algorithm. The GWAS identified the same index variant, SNP1, but with reduced significance (rs1147402472, SAIGE *p* = 3.19 × 10^−7^), indicating a stable result but with a loss of power when continuous variables were examined. Given this across GWAS support, the results presented are from the AI-LSTM binary polygenic mixed model GWAS.

### Opposite haplotypes influence diagonal coupled gait type in CPHs

LD analysis of the lead SNPs (SNP1 and SNP2) revealed several variants in LD. The estimated *r*^2^ values between the lead SNPs and those above the suggestive *p* value threshold are listed in Table S6 and Figure 3.

Haplotypes in CPH were inferred based on the 6 SNPs in LD (*r*^2^ ≥ 0.6) with SNP1 (Table S7). Intriguingly, even with the imbalance of horses sampled from each gait (Colombian trocha, *n* = 67; Colombian trot, *n* = 18), there were 7 haplotypes associated with Colombian trot-gaited horses versus 1 haplotype associated with Colombian trocha ones. Focusing on haplotypes observed at greater than 5% frequency in either gait, hap9, carrying SNP1 rs1147402472-T, was most frequently observed in both (*Colombian trocha*, 90%; *Colombian trot*, 51%), while three additional haplotypes carrying rs1147402472-C were observed in *Colombian trot* horses (hap7, hap4, and hap3; Table S7).

Association analysis showed opposing haplotypes with significant effects (*p* < 0.001) on the gait class (Colombian trocha vs. Colombian trot) (Table 5). 53 of 67 Colombian trocha-gaited horses were homozygous for hap9 (carrying SNP1 rs1147402472-T). In contrast, 9 of 17 Colombian trot-gaited horses were carriers for hap7 (carrying SNP1 rs1147402472-C, Table S7).

**Table 5:** Haplotypes with significant effect on Colombian paso horses’ gait class (Colombian trocha*/*Colombian trot)

					Frequencies		
Haplotype	Sequence^a^	Coef	*p*	Sim.*p*	Total	Colombian trot	Colombian trocha
Hap7	CTGATC	−4.78	**<0.01**	**<0.01**	0.07	0.26	0.03
Hap4	CCGATT	−1.67	0.09	**0.02**	0.02	0.06	0.01
Hap9	TCAGCT	5.55	**<0.01**	**<0.01**	0.81	0.49	0.90

Sim.*p* = *p* value adjusted by using 20,000 permutations; significant results are in bold.

Coef. denotes the coefficient, estimated effect of the haplotype on the score of gait class from the GLM model in the haplotype analysis.

a The order of the SNPs is: rs1147402472 (SNP1), rs1136628503 (SNP2), rs396793867 (SNP3), rs1141932866 (SNP4), rs1145581384 (SNP5), and rs1151964115 (SNP6).

In addition, SNP1 alone explained approximately 10% of the gait variance. In contrast, the combined haplotype derived from SNP1 and SNP2 (Table 2) explained over 48% of the gait variance. We note that the SNP1-SNP2 haplotype of T-C was near to fixed in Colombian trocha-gaited individuals, while the T-C and opposite C-T haplotypes were segregating in Colombian trot-gaited horses. While the variance estimates may be subject to potential effect-size inflation typical of discovery cohorts, the pronounced increase in explanatory power, from the SNP1 to the combined haplotypes, strongly supports a complex, multi-variant regulatory architecture within the QTL. In addition, the full 6 SNPs in high-LD haplotype (Table S6) model explained a similar but the reduced proportion of explained variance, approximately 40%.

### Divergent allelic associations at the ECA16 locus distinguish gaited and non-gaited horses

To test if the variants identified here had the potential to influence gaitedness in additional breeds, we assessed the allele frequencies at SNP1 (rs1147402472) and SNP2 (rs1136628503) across a total of 1,859 horses, encompassing 58 breeds (18 gaited and 40 non-gaited). Data included newly genotyped horses (222 horses and 7 breeds; Table 6) and horses drawn from published datasets (1,637 horses, 55 breeds; Table 6; https://www.ebi.ac.uk/eva/ accessed on 2025-09-05; PRJEB23301, PRJEB28306, PRJEB32686, PRJEB47918, PRJEB55177, PRJEB74212, and PRJEB9799). We found two distinct association patterns within the same QTL that modulate gait type in CPHs and other breeds).

**Table 6:** Frequencies of the associated variants for SNP1 (rs1147402472) and SNP2 (rs1136628503) for gait-type QTL in Colombian paso horses across 58 horse breeds classified as gaited or not gaited

Breed	*n* (total)	Frequency SNP1-C	*n* (total)	Frequency SNP2-T	Gaitedness
**Gaited horses**					

Akhal-Teke	4	0.63	4	0.63	glide^a^
Aegidienberger	2	0.00	2	0.00	tolt, pace
American Curly^b^	52	0.11	51	0.24	foxtrot
American Miniature horse^b^	46	0.11	47	0.06	pace
American Saddlebred^b^	54	0.56	33	0.33	harness, pace
American Standardbred	41	0.20	41	0.09	harness, pace
Brazilian Mangalarga Marchador	1	0.50	–	–	marcha
Chinese Mongolian horse	95	0.12	100	0.10	joroo
Colombian paso fino^b^	30	0.35	28	0.02	–
French trotter	10	0.05	10	0.15	harness
Icelandic horse	388	0.26	397	0.13	tolt, pace
Missouri Fox trotter	1	1.00	1	0.00	foxtrot, pace
Morgan^b^	58	0.27	55	0.16	single-foot
Native Mongolian Chakouyi	1	0.00	–	–	pace
Pura Raza Gallega^b^	7	0.14	7	0.07	–
Rocky Mountain^b^	4	0.25	4	0	–
Tennessee Walking horse	4	0.00	3	0.00	running walk
Welsh pony	22	0.16	22	0.30	pace
Total gaited horses	**820**	**0.26**	**805**	**0.14**	–

**Non-gaited horses**					

American Paint horse	4	0.00	4	0.50	not gaited
Arabian horse	23	0.00	22	0.11	not gaited
Austrian warmblood	1	0.00	1	0.00	not gaited
Badenwürttem-bergisches Warmblut	1	0.00	1	0.50	not gaited
Bayrisches Warmblut	1	0.00	1	0.00	not gaited
Belgian warmblood	2	0.25	2	0.00	not gaited
British warmblood	5	0.00	5	0.40	not gaited
Clydesdale	19	0.13	19	0.11	not gaited
Connemara	4	0.50	4	0.13	not gaited
Czech Republic warmblood	1	0.00	1	0.00	not gaited
Danish warmblood	1	0.00	2	0.25	not gaited
Dutch warmblood	4	0.13	4	0.13	not gaited
Franches-Montagnes	60	0.28	60	0.00	not gaited
French warmblood	37	0.12	37	0.07	not gaited
German Riding pony	2	0.25	2	0.50	not gaited
German warmblood	114	0.09	114	0.26	not gaited
Haflinger	11	0.36	11	0.00	not gaited
Hannoveraner	5	0.00	5	0.20	not gaited
Holsteiner	11	0.09	11	0.41	not gaited
Irish warmblood	2	0.00	2	0.00	not gaited
Japanese Thoroughbred horse	368	0.05	370	0.29	not gaited
Koninklijk Warmbloed Paard	1	0.00	1	0.00	not gaited
Latvian warmblood	1	0.50	1	0.00	not gaited
Lipizzaner	4	0.00	4	0.13	not gaited
Noriker	1	0.00	1	0.00	not gaited
Norwegian Fjord	1	0.00	1	0.00	not gaited
Oldenburger	5	0.00	5	0.60	not gaited
Percheron	4	0.25	3	0.00	not gaited
Polish warmblood	2	0.00	2	0.50	not gaited
Pure Spanish breed	1	0.00	1	0.00	not gaited
Quarter Horse	55	0.16	55	0.23	not gaited
Russian warmblood	1	0.00	1	0.00	not gaited
Shetland pony	23	0.13	23	0.00	not gaited
Sorraia	1	0.50	1	0.00	not gaited
Swedish warmblood	2	0.00	2	0.25	not gaited
Swiss warmblood	214	0.20	214	0.17	not gaited
Thoroughbred	29	0.02	29	0.31	not gaited
Trakehner	5	0.00	5	0.60	not gaited
Westphalian	4	0.50	4	0.38	not gaited
Yakut	9	0.06	9	0.06	not gaited
Total non-gaited horses	**1,039**	**0.12**	**1040**	**0.22**	–

a Classified as gaited horse breed according to a previous work.^20^

b These samples were also genotyped directly with samples available in Dr. Lindgren’s lab and merged with EVA database. The Colombian Paso fino, Pura Raza Gallega, and Rocky Mountain samples only came from Dr. Lindgren’s lab.

The SNP1 rs1147402472-C was found at higher frequencies in horse breeds capable of performing alternate gaits (χ^2^ = 118.5, *n* = 1,859, df = 1, *p* < 2.20 × 10^−16^), such as the diagonal gaited Colombian trot (*n* = 17; frequency (F) = 0.44), lateral gaited CPF (*n* = 30; F = 0.35), and lateral gaited American Saddlebred (*n* = 52; F = 0.58). In contrast, the rs1147402472-C allele was rare in the diagonal gaited Colombian trocha horses, while the rs1147402472-T allele predominated (*n* = 67; F = 0.94) (Tables 3 and 6). To account for the inherent population structure within our meta-analysis dataset, we used a Bayesian generalized linear mixed model (BGLMM). This analysis revealed a notable association between the gaitedness and the presence of the SNP1-C, with an estimated odds ratio (OR) of 2.29 (95% CI: 0.97–5.38). Although the association reached marginal statistical significance using the BGLMM model (*p* = 0.057), the substantial effect size suggests that the SNP1-C-allele is strongly linked to the gaitedness across diverse horse breeds.

SNP2 rs1136628503-T allele was more prevalent in non-gaited populations (χ^2^ = 36.48, *n* = 1,845, df = 1, *p* < 1.57 × 10^−9^), although it was seen to segregate in diagonal gaited *Colombian trot* horses (*n* = 17; F = 0.32) and to a smaller degree in lateral gaited Icelandic horses (*n* = 388; F = 0.13) (Tables 3 and 6). In contrast, the SNP2 rs1136628503-C showed a strong association with both diagonal gaited Colombian trocha (*n* = 67; F = 0.97) and lateral gaited paso fino horses (F = 0.98). Despite these raw frequencies, the BGLMM, which accounts for breed-specific variance, rendered the association between SNP2 and gaitedness non-significant (*p* = 0.322). This suggests that while SNP1-C effectively tags alternate gait phenotypes, the distribution of SNP2-T likely reflects a distinct haplotypic background associated with conventional trot and gallop patterns rather than a primary causal link to gaitedness itself.

### Cross-breed haplotypic analysis reveals ancestral and gait-specific variants across 10 external horse breeds

We extended the two- and six-SNP CPH haplotype analysis (Table 5) to 3 gaited and 7 non-gaited breeds, using public data from the European variation archive (EVA) (Tables S8 and S9). There were between 28 and 397 individuals per breed tested, and from these, we identified 11 6-SNP haplotypes with a frequency above 5% in any one breed (Table S9). Notably, in this 10-breed set, we did not find any copies of the haplotype found at 26% in horses with Colombian trot gait (CTGATC; SNP1-SNP2- … -SNP6; see Table 5 for SNP identifiers). In contrast, the TCAGCT haplotype observed in 49% of Colombian trot gaited-horses and 90% of Colombian trocha gaited horses was observed in all horse breeds tested (range, 35%–67%), suggesting that this represents the ancestral haplotype.

### A subset of QTL genes are linked to locomotion and motor control and show co-expression and interactions

Functional annotation of genes within the gait-associated QTL (Figure S3; Table S5) revealed that most have known functions, with unclassified genes significantly under-represented across all ontologies (*p <* 0.001). While many genes are involved in general processes such as metabolism and biological regulation, a subset shows direct relevance to locomotor function. Notably, *SRGAP3*^21^ and *ATP2B2*^22^^,^^23^ are annotated to regulation of locomotion^19^*, SETD5* is linked to cognition and learning,^24^^,^^25^ and *LHFPL4* contributes to neurotransmission and motor behavior,^26^ suggesting potential roles in coordinated gait. Additional genes associated with response to stimulus (*BRPF1*, *OGG1*, *IRAK2*, *OXTR*, *ATG7*, *HRH1*, *FANCD2*, *CPNE9*, and *RAD18*) may influence sensory input and adaptive motor control, which are critical for precise limb coordination. Together, these results indicate that within a broadly metabolic and regulatory genetic background, specific genes in the QTL region likely modulate gait phenotypes in CPHs, providing a focused set of candidates for future functional studies.

Gene-gene interaction analysis using GeneMANIA^27^ identified several types of functional relationships among the genes within the 2.43 Mb region of interest. The network of 53 genes, including 20 related genes and 224 total links displayed a complex network with co-expression (72.30%), shared protein domains (12.72%), predicted interactions (7.42%), and co-localization (7.29%) (Figure S4). Within this network, we detected a predicted interaction between *ATP2B2* and *SGRAP3*, as well as between *ATP2B2* and *OXTR*. In addition, *ATP2B2* and *SGRAP3* were found to be co-expressed in humans and predicted interaction in mice, further supporting a potential functional relationship between these candidate genes.

## Discussion

This study provides insights into the genetic regulation of gait diversity in CPHs, identifying a 2.43 Mb QTL associated with gait type in horses homozygous for the wild-type (non-gaited) *DMRT3* allele. By integrating AI-driven gait classification with high-density genomic analysis, we highlight *ATP2B2* and *SGRAP3* as potential candidate genes influencing limb coordination and neural function. These findings provide evidence for the genetic architecture underlying alternative gaits and offer a foundation for future research on coordinated locomotion in mammals.

### Objective measurements and machine learning allow gait classification in CPHs

One of the most significant challenges in studying the CPH gaits is accurately classifying them. While Colombian trocha and Colombian trot are distinct gaits, they share several similarities, including fast locomotion and high stride frequencies.^17^ These similarities often result in misclassification, especially when grouping is based solely on visual or auditory cues, which are inherently limited by human perceptual capabilities. In this study, AI machine learning techniques, specifically AI-LSTM neural network algorithm, were employed to objectively classify the gait of horses based on IMU sensor motion data. This approach revealed a significant discrepancy between traditional classifications made by breeders and the machine learning model’s classifications. Notably, compared with the breeder’s reports, the algorithm classified 58% more horses as performing Colombian trocha gait and 45% fewer horses as performing Colombian trot gait (Figure S1). This indicates that the traditional methods based on visual assessment and subjective interpretation are prone to biases. The effectiveness of machine learning in gait classification underscores the potential of AI-LSTM and quantitative tools to improve breeding practices and gait-related studies, offering objective and repeatable assessments crucial for preserving the genetic integrity of distinct gaits, such as Colombian trocha and Colombian trot.

### The GWAS reveals genetic differences between Colombian trocha and Colombian trot

The genomic analysis of the CPHs revealed a significant QTL on ECA16 associated with the distinction between Colombian trocha and Colombian trot gaits. This discovery helped identify a significant SNP in this region (ECA16:8860175, rs1147402472) that was found to be consistently associated with gait type across multiple models. This association supports the hypothesis that Colombian trocha and Colombian trot are phenotypically different gaits and show genetic differences, rather than variations of the same gait as previously suggested.^28^

### Contrasting haplotypes and their role in gait differentiation

LD analysis within the QTL span was used to classify haplotypes for further analysis. Here, we identified several SNPs in LD close to the SNP1 (Table S6). The haplotypes estimated from SNP1 (intergenic, ECA16:8860175, rs1147402472) revealed that there were multiple haplotypes that segregated with the Colombian trot gait (Figure 3; Table S7), and some of these showed significant effects (*p* < 0.001) on gait type (hap9: TCAGCT for Colombian trocha and hap7: CTGATC for Colombian trot) (Table 5), which is particularly noteworthy.

In addition, the observation that the haplotypes based only on the SNP1 or SNP2 account for 48.6% of the gait variance suggests that the main association signal is captured by these two lead SNPs. The inclusion of additional 4 SNPs in LD with the SNP1 and SNP2 did not enhance the explanatory power, but rather diluted the association, likely due to haplotype fragmentation without introducing novel biological signal. Therefore, the localized haplotype defined by the two SNPs, SNP1 and SNP2, provides the most robust representation of the genetic contribution to gait variance. Furthermore, the six SNPs define multiple distinct haplotypes associated with Colombian trot gait and collectively delimit a genomic region of high interest. Taken together, these results suggest that key genetic factor(s) in or near this region may influence gait characteristics in CPHs.

The identification of these opposing haplotypes provides information relevant to the classification of CPH gaits, particularly Colombian trot. The high frequency of the Colombian trot gait*-*associated haplotype (hap7: CTGATC, F = 0.26) and the practical absence of homozygous horses for this haplotype (one of the 85 horses analyzed) could be explained by a negative selection against this haplotype, and/or a favoring selection for the hap9 (TCAGCT) associated with Colombian trocha gait. The Colombian trocha-gaited horses have gained popularity over the other gaited horses (paso fino and Colombian trot) in the last 20 years. In 2005, 32.12% of the registered CPHs were classified as Colombian trocha gaited, increasing to 39.78% in 2019, based on studbook records (https://www.fao.org/dad-is/). In addition, over the last decades, breeders have increasingly selected Colombian trocha horses with higher stride frequencies, and some have begun crossing Colombian trocha- and Colombian trot-gaited horses with the aim of increasing stride frequency in the Colombian trot gait as well. These breeding practices have contributed to a gradual loss of clarity of the footfall pattern and other defining characteristics of Colombian trot-gaited horses.

Proactive measures, including objective classification, selective breeding, and dedicated conservation efforts, are recommended to preserve the prevalence of the Colombian trot gait*-*associated haplotype (CTGATC).

### Fine-scale genetic variation at ECA16 modulates neural and biomechanical control of gait

The identification of divergent allelic and haplotypic patterns at the ECA16 locus provides a plausible genetic basis for the fine-scale coordination differences observed between CPH gaits. Our findings indicate that individuals from gaited breeds are 2.29 times more likely to carry the SNP1 rs1147402472-C allele than those from non-gaited breeds; notably, this association persists even after rigorously accounting for breed-specific genetic backgrounds through Bayesian mixed-effects modeling. This suggests that this QTL contains conserved variants that confer the ability to perform alternate gaits across diverse equine populations.

Specifically, haplotypes carrying the SNP1-C allele (either in the CC configuration or the infrequent CT variant except in Colombian trot gait) were predominantly identified in gaited lineages, appearing to tag a genetic signature associated with lateral or alternate gaits. In contrast, the TT haplotype (SNP1-T and SNP2-T) was most common in non-gaited breeds, characterizing lineages traditionally involved in racing or conventional equestrian activities. Given the neurobiological roles of the candidate genes within this region, these variants likely influence the neural circuits governing motor learning, neuromuscular coordination, and postural control—mechanisms fundamental to maintaining the rhythm and balance required for specialized locomotion.

The contrasting haplotype associations and the specific linkage of the SNP1-C with gaited phenotypes, together with the total absence of the Colombian trot gait hap7 (CTGATC, Table 5) across 10 external breeds (Table S9), underscore a complex allelic architecture within the ECA16 region. This pattern suggests that CPHs may harbor a unique regulatory or coding variant, or a specific allelic combination, that collectively accounts for a substantial proportion of the observed variation in diagonal gait expression.

Overall, these findings indicate that fine-scale genetic differentiation at the ECA16 locus likely modulates the vestibular or neurological pathways essential for the neural and biomechanical coordination that distinguishes gaited from non-gaited locomotion. Our results suggest that the ECA16 QTL region has undergone selective refinement under breed-specific pressures to achieve precise motor and postural control, exerting a regulatory influence on diagonal gaits.

### Interpretation and implications of candidate genes’ function within the QTL region

Further investigation into the candidate genes within the identified QTL region revealed two strong candidate genes based on their function and location. The first gene is *SRGAP3.* This gene has been implicated in cytoskeletal reorganization during brain development to control processes such as synaptic plasticity.^21^ *SRGAP3*-knockout mice exhibited neurodevelopment disorders, including lower locomotor activity in males.^21^ A previous study of horse bulk RNA sequencing data supports a similar role for this gene, showing higher expression in the cerebellum and embryo than in other tissues such as articular cartilage, synovial membrane, placental villi, and testes.^29^ These facts suggest that *SRGAP3* is a candidate gene for further study in relation to complex locomotion patterns.

*ATP2B2*, supported by the intronic SNP2 rs1136628503, emerges as a second strong candidate gene. This gene has a well-established role in neuromuscular and sensory functions, and it encodes the plasma membrane Ca^2+^ ATPase isoform 2 (PMCA2), a protein essential for calcium homeostasis in neurons and in the sensory hair cells of the inner ear. *ATP2B2* has been implicated in brain morphogenesis, neurodevelopment, hearing, balance, and motor coordination. In mice, coding mutations alter cerebellar morphology,^22^^,^^23^ disrupt inner ear structure,^23^^,^^30^^,^^31^^,^^32^^,^^33^^,^^34^^,^^35^^,^^36^^,^^37^ and impair locomotor performance,^22^^,^^23^^,^^31^^,^^36^^,^^38^ producing phenotypes such as ataxia, gait abnormalities, impaired limb coordination, and loss of balance. In humans, pathogenic variants—mostly coding—are associated with hearing loss.^36^^,^^37^^,^^38^ Although hearing loss and locomotor deficits can occur independently, experimental and clinical studies indicate that dysfunction of the inner ear often affects both systems because the auditory and vestibular receptors are anatomically and functionally intertwined within the same organ.^39^^,^^40^ The vestibular system is the primary sensor for balance and postural control; thus, perturbations affecting inner ear structure or calcium signaling can manifest as both auditory and gait-related phenotypes. For horses, no obvious hearing-related abnormalities were noted in the animals included in this study, although specific audiological testing would be required to confirm this, and there is currently limited evidence linking *ATP2B2* variation to equine hearing or balance phenotypes.^39^ Expression data further support its functional relevance: *ATP2B2* is abundantly expressed in nervous tissue in humans^41^ and mice,^42^^,^^43^ with particularly high levels the mouse cerebellum.^44^

Given this expression pattern, especially in cerebellar Purkinje cells in mice (Figure S5), considering that the cerebellum is the primary center for motor coordination, “fine-tuning” the timing of muscle contractions, and maintaining balance, and the gene’s role in calcium regulation, *ATP2B2* is a plausible candidate gene influencing fine motor coordination. Because Purkinje cells are central to the timing and modulation of motor outputs, even subtle changes in *ATP2B2* activity could influence cerebellar circuit function and, by extension, gait characteristics. This raises the intriguing possibility that gait differences, such as those observed in Colombian trocha-gaited versus Colombian trot-gaited horses, may be partly influenced by the functional or regulatory variation in *ATP2B2.*

Interestingly, *ATP2B2* expression is several times higher than that of *SGRAP3* in the cerebellum, and both genes are co-expressed in humans.^45^ Our gene-gene interaction analysis also suggests a potential functional connection between *ATP2B2* and *SGRAP3*. Although the precise biological implications in horses remain to be determined, co-expression of calcium-handling genes (such as *ATP2B2*) with regulators of neuronal morphology (such as *SGRAP3*) has been associated with motoneuron plasticity and the refinement of neuronal circuits.^46^ Previous work^47^ shows that proteins involved in dendritic spine and filopodia dynamics contribute to motor-neuron development, synaptic stabilization, and adaptive motor control.^47^ If *ATP2B2* and *SGRAP3* participate in similar processes in horses, their combined activity could influence the coordination of limb movement, balance, and neural control required for different gait patterns.

The most likely scenario is that the Colombian trocha gait-associated variant could slightly reduce the efficiency of the Ca^2+^ pump encoded by *ATP2B2*, resulting in slower calcium clearance and prolonged intracellular Ca^2+^ transients following neuronal activity. Such a change could subtly alter the recovery dynamics of locomotor neurons within the spinal circuitry that controls rhythmic movement. Even small changes in calcium decay kinetics can influence burst timing and phase relationships in central pattern generator networks, potentially destabilizing the strict left-right synchrony required for the four-beat Colombian trot gait. This could facilitate the emergence of a slight shift in the limb coordination pattern, allowing the transition toward the four-beat Colombian trocha gait.

The following closest genes (in order) to the SNP1 are *THUMPD3*, *RAD18*, *SETD5*, *LHFPL4*, and *MRTM14* (Figure 3B). *THUMPD3* is involved in tRNA methylation. *RAD18* is involved in fertility,^48^ oxygen consumption related to hematocrit and hemoglobin levels, and corpuscular volume (the average size of the red blood cells),^49^ affecting the performance and locomotion execution differences of these gaits. *SETD5* (linked to cognition and learning^24^^,^^25^) suggests that cognitive and sensory integration may also contribute to the fine-tuning of gait patterns. Similarly, *LHFPL4* is associated with neurotransmission, and motor behavior^26^ may play a direct role in the neural control of gaits. *MTMR14* is involved in the homeostasis of Ca^+2^ in muscles, affecting limb coordination in mice.^50^

Additional annotated genes within the 2.43 Mb QTL region (Table S5) are associated with neurotransmission and neurodevelopment (*BRPF1*^51^^,^^52^*, OGG1*,^53^ and *OXTR*^54^), embryonic development (*TADA3*^55^), anemia (*FANCD2*^56^ and *FANCD2OS*^56^), eye structure (*TTLL3*,^57^ *EMC3*,^58^ and *TATDN2*^59^), lipid metabolism (*CIDEC*,^60^ *EMC3*,^61^ *TATDN2*,^62^ and *GHRL*^63^), body weight (*RPUSD3*^64^ and *GHRL*^65^), metabolism and energy homeostasis (*GHRL*^66^^,^^67^), immunodeficiency disorders (*JAGN1*,^68^ *IL17RE*,^69^ *IL17RC*,^70^ and *IRAK2*^71^^,^^72^), heart development (*CRELD1*^73^), and behavior (*OXTR*^74^^,^^75^) but these are not directly associated with locomotion traits.

Collectively, these findings suggest that gait-related genetic variation in this region may involve genes with roles in neuromuscular function, synaptic plasticity, and cytoskeletal organization. While a subset of these candidate genes align with physiological processes critical for locomotion, further functional studies, such as gene expression profiling and experimental validation, are necessary to confirm their roles in gait determination.

### Candidate gene enrichment aligns with known physiological processes underlying gait traits

The functional classification of candidate genes provides insight into the biological processes that may underlie gait variation in CPHs. Although many genes mapped to general categories such as cellular metabolism and regulation, the presence of genes specifically linked to locomotion (*SGRAP3*) and anatomical structure regulation (*OXTR*) suggests that gait diversity may be shaped by a subset of genes with precise roles in motor control. Similarly, genes associated with response to stimulus could reflect neural and sensory mechanisms essential for adaptive locomotion and coordination. These findings align with our gene-gene interaction results and highlight that, rather than broad pathways, specialized molecular and neural processes may be involved in the subtle differences in gait performance. This perspective provides a mechanistic context for how specific candidate genes within a QTL may contribute to coordinated locomotion in mammals.

### AI-enabled genomic mapping identifies a major ECA16 QTL driving fine-scale locomotor differentiation in horses

This study provides a comprehensive genomic and kinematic framework for the differentiation of complex locomotor phenotypes in the CPHs. We objectively characterized the subtle biomechanical distinctions between the gait types Colombian trocha and Colombian trot by using machine learning-based approach, and by integrating this AI-based gait classification with high-density genomic mapping, we identified a major QTL on ECA16. Our findings reveal that fine-scale genetic differentiation at this locus, marked by divergent, breed-specific haplotypes, likely modulates the vestibular and neurological pathways essential for the precise motor and postural coordination that distinguishes gaited from non-gaited locomotion. Notably, this regulatory influence on diagonal gait signatures extends beyond the well-documented effects of the *DMRT3* “gait keeper” mutation.

The success of the AI-LSTM neural network in resolving these phenotypes offers a robust tool for the objective preservation of specialized gaits and the refinement of breeding strategies. Future research should prioritize whole-genome sequencing and functional validation of candidate genes within the ECA16 QTL to further elucidate the molecular mechanisms governing high-frequency locomotor coordination. Ultimately, these results provide a template for using integrative AI-enabled genomic workflows to study the evolution of coordinated movement, with implications for understanding the fundamental principles of mammalian locomotion.

### Limitations of the study

One limitation of this study is the relatively small sample size of genotyped horses included in the GWAS, which may impact the robustness and generalizability of the identified associations. While the current study provides valuable insights into the genetic underpinnings of gait differences in CPHs, further research with a larger cohort of horses is necessary to confirm and refine these findings. To address this, we are currently sequencing and phenotyping additional horses to validate and expand upon the initial results to provide a more comprehensive understanding of the genetic architecture of gait traits in CPHs. Moreover, while the use of machine learning in this study facilitated the analysis of complex datasets, the genetic architecture underlying gait differences remains unresolved. Without leveraging machine learning, addressing these complexities would have been significantly more challenging, underscoring its utility despite the remaining challenges.

## Resource availability

### Lead contact

Requests for further information and resources should be directed to and will be fulfilled by the lead contact, Miguel Novoa-Bravo (miguelnovoa@geancol.com).

### Materials availability

This study did not generate new unique reagents.

### Data and code availability

• Genotyping array data and metadata have been deposited at European Variation Archive (EVA): PRJEB105194, and are publicly available as of the date of publication.
• Phenotype data have been deposited in Mendeley Dataset: https://doi.org/10.17632/52rzdhx297.1
• This paper does not report original code.
• Any additional information required to reanalyze the data reported in this paper is available from the lead contact upon request.

## Acknowledgments

We thank Mr. Héctor Barriga for the support in the slow-motion visual analyses and valuable comments and Dr. Rakan Naboulsi for valuable comments. We also thank Dr. Marina Solé and Dr. Juan Cordero for their support in the initial genomic analyses and their comments. We also thank Fedequinas, particularly the CPH breeders who participated in this study. This study was funded by The 10.13039/501100004359Swedish Research Council (VR) to G.L.; the Swedish Research Council for Sustainable Development (FORMAS) to G.L.; 10.13039/501100003792Swedish Brain Foundation (FO2022-0018) and Olle Engkvist Byggmästare Foundation (220-0254) to K.K.; 10.13039/501100004359Swedish Research Council (2022-01245) to K.K.; European Master in Animal Breeding and Genetics (EMABG) to B.V.D.V.; Genética Animal de Colombia SAS to M.N.-B.

## Author contributions

Conceptualization, M.N.-B. and G.L.; data curation, M.N.-B. and B.V.D.V.; methodology and investigation, M.N.-B. and G.L.; formal analysis, M.N.-B. and J.R.S.M.; resources, G.L., M.R., and M.N.-B.; software, F.S.-B. and M.N.-B.; funding acquisition, G.L., K.K., and M.N.-B.; visualization, M.N.-B.; writing – original draft, M.N.-B., J.R.S.M., and G.L.; writing – review & editing, M.N.-B., J.R.S.M., G.L., F.S.-B., K.K., and M.R.

## Declaration of interests

The authors declare competing interests concerning the commercial applications of the current study. G.L. is a co-inventor of a patent application concerning commercial testing of *DMRT3* mutation. The stated patent does not restrict research applications of the method. G.L. is also a shareholder and CEO of Capilet Genetics, Inc., and M.N.-B. is a shareholder of Genética Animal de Colombia SAS.

## Declaration of generative AI and AI-assisted technologies in the writing process

During the preparation of this work, the author(s) used ChatGPT/Gemini to improve the readability and language in some paragraphs of the article. After using this tool/service, the author(s) reviewed and edited the content as needed and take(s) full responsibility for the content of the published article.

## STAR★Methods

### Key resources table

**Table:** 

REAGENT or RESOURCE	SOURCE	IDENTIFIER
**Biological samples**		

Horse follicles hair samples (discovery cohort)	This paper	N/A
Horse follicles hair samples (external cross-validation cohort)	Promerová et al.^11^ Lindgren Lab resources	N/A

**Critical commercial assays**		

Custom TaqMan™ SNP Genotyping Assay (non-human)	ThermoFisher	Cat#4332077
Axiom™ Equine Genotyping Array (670K)	ThermoFisher	Cat#550583

**Deposited data**		

Phenotype data	Mendeley data	https://doi.org/10.17632/52rzdhx297.1
Genotyping array data and metadata for Colombian Paso Horses	European Variation Archive (EVA)	https://wwwdev.ebi.ac.uk/eva/ Accession number: PRJEB105194
Multi-breed validation SNP datasets	European Variation Archive (EVA)	https://www.ebi.ac.uk/eva/ (accessed on 2025-09-05) Accession numbers: PRJEB23301, PRJEB28306, PRJEB32686, PRJEB47918, PRJEB55177, PRJEB74212, and PRJEB9799

**Oligonucleotides**		

*DMRT3* genotyping assay primers and probes: Forward primer: CCTCTCCAGCCGCTCCT; reverse primer: TCAAAGATGTGCCCGTTGGA; wild-type probe: CTGCCGAAGTTCG; mutant probe: CTCTGCCTAAGTTCG. SNP1 genotyping assay primers and probes: Forward primer: CTTTATGTGCAGGATTTGAGG; reverse primer: CCTCCTTTCTTTCTCCAGGGC; wild type probe: TGTGCAGGATTTGAG; mutant probe: TGCAGGATTTGAGGC. SNP2 genotyping assay primers and probes: Forward primer: TGCGATGCCTGTGAGAAATCT; reverse primer: CCAGCCTGGACCATTGTATCC; wild type probe: CTCGACACGTCCTCTT; mutant prob: CTCGACACATCCTCTT.	ThermoFisher	Cat#4332077

**Software and algorithms**		

Inertia Studio	Bosch^76^	https://inertia-technology.com
MATLAB 2018b	MathWorks, Natick, Massachusetts, USA	https://www.mathworks.com
RWizard Software v.1.1.	Guisande et al.^77^	https://webhook.sglaeonline.com/
R v.3.3.3	R Foundation for Statistical Computing	https://www.r-project.org/
Rstudio v.1.0.153	Posit Software, PBC, Boston, MA. URL	http://www.posit.co/.
GenABEL v.1.8	Karssen et al.^78^	https://cran.r-project.org/src/contrib/Archive/GenABEL/
GCTA v.6.1	Yang et al.^79^	https://github.com/JianYang-Lab/GCTA
SAIGE v.1.4.5	Zhou et al.^19^	https://github.com/saigegit/SAIGE
PLINK v1.9	Purcell et al.^80^	www.cog-genomics.org/plink/1.9/
GeneMANIA	Mostafavi et al.^27^	https://genemania.org/
PANTHER v.19.0	Muruganujan et al.^81^	https://pantherdb.org
Biorender	Biorender	https://Biorender.com.

### Experimental model and study participants details

#### Discovery cohort and animal characteristics

A primary discovery cohort comprising 225 Colombian Paso horses (*Equus ferus caballus*) (consisting of 100 stallions/geldings and 125 mares aged 36–120 months) was assembled for kinematic and genomic analysis. Horses were sourced from 62 horse farms across eight Colombian departments: Antioquia, Boyacá, Caldas, Cundinamarca, Córdoba, Risaralda, Sucre, and Valle del Cauca. Inclusion criteria required a balanced sex ratio and a representative distribution of breeder-classified gaits (Colombian trocha vs. Colombian trot). Additionally, eight Colombian Paso Fino horses were included as an outgroup control to ensure the robustness of the gait classification algorithm.

#### External cross-validation cohort

To validate the generalizability of identified markers, an external cross-validation cohort (*n* = 222) was established using hair follicle samples from seven distinct gaited breeds. This cohort included: American Miniature Horse (*n* = 41), American Saddlebred (*n* = 52), American Curly (*n* = 50), Colombian Paso fino (*n* = 30), Rocky mountain (*n* = 5), Morgan (*n* = 38), and Pura Raza Gallega (*n* = 7). Samples were curated from previously published datasets^11^ and supplemental unpublished resources from the Lindgren Laboratory (SLU-Sweden).

#### *In silico* meta-analysis dataset

In addition, to validate the generalizability of identified markers aproximately 1,650 horse genotypes across 55 breeds from European Variation Archive (EVA), (https://www.ebi.ac.uk/eva/accessed on 2025-09-05) were obtained with the same porpoise.

#### Pedigree analysis and kinship minimization

To minimize genetic redundancy, pedigree data provided by Fedequinas (the national federation for the breed) were utilized to select individuals with the lowest possible kinship coefficients. The reference pedigree database consisted of approximately 220,000 records, encompassing four primary gait categories: Colombian trocha horses (21%), Colombian trocha and trot horses (5%), Colombian trot and gallop horses (46%), and Colombian Paso Fino horses (28%). This database, which included comprehensive parentage, birth dates, gait classification, and sex records, was analyzed as previously described.^82^

#### Ethical statement

The Ethics Committee for Animal Experiments in Uppsala, Sweden approved the experimental protocol for the dataset with permit number 5.8.18–05055/2019. All the methods were carried out on each horse under the approved guidelines and regulations. We obtained informed consent from the breeders for each animal. All data were from sound horses according to the breeders.

### Method details

#### Kinematic data acquisition via IMU sensors

Objective kinematic data were collected from 225 horses between 2018 and 2020 using a wireless network of seven Inertial Measurement Units (IMUs; ProMove-mini, Inertia Technology, Enschede, The Netherlands).^76^ Sensors were securely affixed to the poll, withers, pelvis (sacrum), and the dorsal aspect of all four limbs, following previously validated protocols.^28^ Data were recorded while horses were ridden over straight, hard-surface segments for durations of 3–5 min. Movements were performed at gaits designated by the rider or owner, including walk, paso fino, Colombian trot, Colombian trocha, and Colombian gallop.

#### Data preprocessing and video synchronization

To ensure precise gait identification, all IMU data streams were time-synchronized with high-definition video recordings. Expert evaluators specializing in Colombian Paso Horse (CPH) locomotion performed slow-motion video analysis to isolate straight, regular, and representative locomotion sequences for downstream analysis. Stride parameters were extracted from these curated raw IMU segments using a validated gait-analysis algorithm.^83^ To maintain high data quality, kinematic variables were subjected to outlier detection and removal on an individual horse basis prior to neural network integration.

#### AI-LSTM neural network training and validation

The processed kinematic segments were utilized to train and validate an Artificial Intelligence-Long Short-Term Memory (AI-LSTM) neural network architecture.^28^ The classification algorithm was trained using a “gold standard” reference cohort of 39 horses (14 Colombian trot and 25 Colombian trocha). The gait phenotype for each reference horse was verified via consensus by CPH experts through slow-motion video inspection, ensuring strict adherence to the locomotor standards established by the national breed authority, Fedequinas. This AI-driven approach allowed for a high-resolution, objective reclassification of the broader study population.

#### Genomic DNA extraction

Genomic DNA was isolated from hair follicle samples of 217 Colombian Paso Horses (CPHs) using a modified Chelex-proteinase K extraction protocol.^84^ Briefly, hair bulbs (7–10 for TaqMan assay and 15–20 for array genotyping) were incubated with 100 mL of 5% Chelex 100 Resin (Bio-Rad Laboratories, Hercules, CA, USA) and 7 mL of proteinase K (20 mg/mL; Merck KGaA, Darmstadt, Germany). Samples were incubated at 56°C for 1 h followed by proteinase K inactivation at 95°C for 10 min. The resulting supernatant containing genomic DNA was utilized for all subsequent molecular analyses.

#### *DMRT3* genotyping and cohort selection

To isolate the genetic basis of diagonal gaits from the known major influence of the *DMRT3* “Gaitkeeper” mutation, we performed targeted genotyping of the rs1150690013 nonsense SNP (*DMRT3* g.22999655C>A) for 217 horses (the initial cohort excluding 8 Colombian Paso Fino individuals). Genotyping was conducted using a custom-designed TaqMan SNP Genotyping Assay (Applied Biosystems, Foster City, CA, USA) on a StepOnePlus Real-Time PCR System (Life Technologies) following previously described protocols.^10^ Consistent with our study objective to identify other loci, all individuals carrying the *DMRT3* variant allele (rs1150690013-A) were excluded from downstream genomic investigations.

#### Genome-wide high-density genotyping

A discovery cohort of 95 horses, all confirmed to be homozygous for the wild-type DMRT3 allele (C/C), was selected for high-density genome-wide analysis. Samples were genotyped using the Axiom Equine Genotyping Array (Thermo Fisher Scientific, Santa Clara, CA, USA), which provides approximately 670,000 SNPs. This array allowed for the high-resolution mapping of the QTL while ensuring that the phenotypic variation observed was independent of the *DMRT3* locus.

#### Cross-population validation cohort

To evaluate the generalizability of the identified GWAS signals beyond the discovery cohort, an external validation study was conducted using a cohort of 222 horses representing seven diverse breeds: American Miniature Horse, American Saddlebred, American Curly, Colombian Paso Fino, Rocky Mountain Horse, Morgan, and Pura Raza Gallega. Genomic DNA was isolated from hair follicle samples using a modified Chelex–proteinase K extraction protocol.^84^ Targeted genotyping of the SNP1 and SNP2 variants was performed using custom-designed TaqMan SNP genotyping assays (Applied Biosystems, Foster City, CA, USA).

#### In silico cross-breed meta-analysis

To investigate the broader evolutionary context and allelic architecture of the identified QTL, we assessed the distribution of SNP1 and SNP2 variants and associated loci across a global panel of 55 horse breeds. Genotypic data for the SNP1, SNP2 and 4 additional SNP markers in high linkage disequilibrium (*r*^2^ ≥ 0.6) to SNP1 were retrieved from the European Variation Archive (EVA) (https://www.ebi.ac.uk/eva/; accessed September 5, 2025). This meta-analysis encompassed a comprehensive dataset of 1,637 to 1,650 individual genotypes, depending on site-specific coverage. These data were utilized to determine whether gait-associated haplotypes identified in the Colombian Paso Horse were population-specific or conserved across other specialized gaited populations.

### Quantification and statistical analysis

#### Comparative gait classification

To evaluate the reliability of phenotypic labels, we contrasted two distinct classification methodologies for Colombian trocha and Colombian trot gaits: traditional breeder-based subjective assessment and objective AI-driven classification utilizing raw IMU kinematic data. All data processing, feature engineering, and model training were executed in MATLAB v.2018b (MathWorks, Natick, MA, USA).

#### AI-LSTM neural network and training

We implemented an Artificial Intelligence-Long Short-Term Memory (AI-LSTM) neural network, a recurrent architecture specifically suited for time-series kinematic data.^28^ The AI-LSTM was designed to model the non-linear relationship between high-frequency sensor readings and specific locomotor phenotypes. The model outputs a probability distribution across nine discrete gait classes: walk, trot, left canter, right canter, tölt, pace, paso fino, Colombian trocha, and Colombian trot.

#### Phenotypic assignment and reclassification criteria

Gait probabilities were calculated on a per-stride basis for the entire study population (*n* = 225). A horse was definitively assigned to a gait class if the model’s posterior probability for that class exceeded a 50% threshold. In instances where the probability was split specifically between Colombian trocha and Colombian trot gaits without reaching the 50% threshold for the horse was designated as “misclassified”. This conservative threshold ensured that the final phenotypes used for the Genome-Wide Association Study (GWAS) were based on high-confidence locomotor signatures rather than subjective breeder categories.

#### Descriptive and comparative statistics

Kinematic variables were stratified by AI-defined gait phenotypes to calculate mean, standard deviation (SD), skewness, and kurtosis. Normality was assessed for all parameters using the Shapiro-Wilk test via the shapiro.test function in the R stats package. Significant differences between Colombian trocha and Colombian trot gait variables were evaluated using Student’s *t* test for normally distributed data or the nonparametric exact two-sample Kolmogorov-Smirnov test for non-normal distributions. All univariate analyses were conducted using the StatR package within the Rwizard software environment.^77^

#### Multivariate analysis and PCA

To visualize the divergence between subjective and objective classification methods, Principal Component Analysis (PCA) was performed. A stepwise selection process was implemented to identify the subset of kinematic parameters that best characterized the variance between groups. PCA was executed using the prcomp() function in StatR via Rwizard.^77^ Two-dimensional PCA visualizations were generated using the plot.cancor() function from the candisc package.

#### Genotype quality control and filtering

Quality control (QC) of the high-density 670K genotyping array data was performed using the GenABEL v.1.8 package^78^ using R v.3.3.3 (R Foundation for Statistical Computing) in R Rstudio v.1.0.153 (Posit Software, PBC, Boston, MA.). To ensure high-quality genomic data, markers and samples were filtered based on the following stringent criteria: SNP minor allele frequency (MAF) < 5%, SNP missingness >5%, sample missingness >15%, and two round of pruning, first using Hardy-Weinberg equilibrium removing loci with a *p* < 1 x 10^−10^ and second those exceeding a false discovery rate (FDR) < 20%.

#### Population structure and kinship analysis

To account for potential confounding due to population stratification, an autosomal genomic kinship matrix was constructed. We utilized K-means clustering (K = {1,2, …,10}) within GenABEL^78^ to identify the optimal number of subpopulations. The sum of the within-cluster sum of squares (sum WCSS) was plotted against K, and the first inflection point (the “elbow” method) was used to define K = 3 as the optimal number of clusters.^85^

#### Multi-model GWAS and robustness testing

GWAS were performed using GenABEL v.1.8^78^ and GCTA v.1.6.^79^ Preliminary linear modeling via ANOVA indicated that sex and age had no significant effect on the gait classes (*p* > 0.05); consequently, these variables were excluded as covariates in the final association models. Association signals were evaluated for significance after correcting for population structure and individual relatedness.

To ensure the robustness of identified associations and account for population stratification, genome-wide association studies (GWASs) were performed using both subjective (breeder-based) and objective (AI-LSTM) phenotypic labels. We implemented a polygenic hierarchical generalized linear model (polygenic_hglm) incorporating a genomic kinship matrix within GenABEL v.1.8.^78^ To further validate findings across different statistical frameworks, we employed: 1. A mixed model-structured association approach (mmscore function with the strata option). 2. GRAMMAR-Gamma (Genome-wide Rapid Association using Mixed Model and Regression - Gamma). 3. Principal Component Analysis (PCA) via the EIGENSTRAT method to adjust for latent population structure.

#### MLMA-LOCO and SAIGE analysis

To address “proximal contamination” the inflation of the test statistic when a candidate SNP is included in the genomic relationship matrix (GRM) we executed in GCTA v.1.66^79^ a Mixed Linear Model Association analysis with the Leave-One-Chromosome-Out (MLMA-LOCO) approach and an FDR correction. Additionally, to account for potential case-control imbalance and the binary nature of the AI-LSTM classification, we utilized SAIGE v.1.4.5,^19^ which employs a saddlepoint approximation to provide accurate *p*-values in unbalanced datasets.

Genome-wide significance was established using a Bonferroni-corrected threshold of *p* < 1.3 × 10^−7^ (calculated based on the total number of SNPs post-QC). A “suggestive” significance threshold was defined at *p* < 1.0 × 10-5. Manhattan and regional association plots, color-coded by pairwise linkage disequilibrium (*r*^2^), were generated using the cgmisc v.2.0 package in Rstudio (v.1.0.153 Posit Software, PBC, Boston, MA.).

#### Haplotype construction and association

To characterize the local genomic architecture of the identified QTL, we calculated pairwise linkage disequilibrium (*r*^2^) for the two significant SNP1 and SNP2 and all variants within a 1-Mb window using PLINK v.1.9.^80^ Haplotype analysis was executed using the haplo.stats package in Rstudio (v.1.0.153 Posit Software, PBC, Boston, MA.). We estimated haplotype frequencies via the Expectation Maximization (EM) algorithm using the haplo.em() function, incorporating variants in strong LD (r^2^ ≥ 0.6) with the lead signals.

The association between specific haplotypes and gait phenotypes was modeled using a generalized linear model via the haplo.glm() function. To ensure statistical stability, the most frequent haplotype was designated as the reference, and only those with a frequency >0.02 were included in the final model. Significance was determined through 100,000 permutations assuming an additive genetic effect.

#### Estimation of explained phenotypic variance

We quantified the proportion of phenotypic variation explained by the SNP1 and SNP2 genotypes and haplotypes using a polygenic hierarchical generalized linear model (polygenic_hglm()) within GenABEL.^78^ Additionally, given the binary nature of the AI-LSTM classification, M McFadden’s Pseudo-R^2^ was utilized to estimate the variance explained by the identified haplotypes. This was calculated as R^2^ McFadden = 1−(Residual Deviance/Null Deviance). These values were derived from the haplo.glm() output to assess the relative contribution of each locus to the distinct diagonal gait signatures.

#### Cross-validation: Comparative frequency analysis

For both the external validation cohort (*n* = 222) and the *in silico* meta-analysis dataset (*n* ∼1,650 across 55 breeds from European Variation Archive (EVA), (https://www.ebi.ac.uk/eva/accessed on 2025-09-05), allelic and haplotypic frequencies were determined via direct counting across all included breeds. To evaluate the association between gaitedness (defined as the presence of specialized diagonal gaits) and the SNP1 and SNP2, Chi-square tests were performed using the VIII2 function within the StatR package in Rwizard v.1.1.^77^ To account for breed effect in the meta-analysis, we employed a Bayesian Generalized Linear Mixed Model (BGLMM) on the filtered dataset (*n* > 5 individuals per breed) controlling for the effect of breed as a random variable using the bglmer() function. In addition, the effect size of the association between gaitedness and the target allele was quantified using Odds Ratios (OR). These values were calculated by exponentiating the fixed-effect coefficients (β) estimated from the Bayesian Generalized Linear Mixed Model (BGLMM), fitted in R v.3.3.3 (R Foundation for Statistical Computing) using RStudio v.1.0.153 (Posit Software, PBC, Boston, MA). This analysis assessed whether specific alleles were significantly enriched in breeds characterized by specialized locomotor phenotypes compared to non-gaited controls.

#### Cross-validation: Haplotype estimation

To achieve high-resolution validation of the identified QTL, a subset of horse breeds with a sample size of *n* ≥ 29 was selected for population-specific haplotype estimation using the *in silico* meta-analysis dataset. Haplotype frequencies within these independent populations were calculated using the Expectation Maximization (EM) algorithm via the haplo.em() function in the haplo.stats package. All computational procedures were executed in RStudio v.1.0.153 (Posit Software, PBC, Boston, MA, USA). This threshold ensured sufficient statistical power to confirm the conservation of the gait-associated haplotypes across diverse genomic backgrounds.

#### Gene-gene interaction analysis

Candidate genes were identified based on the EquCab 3.0 reference genome (GCA_002863925.1) using the Ensembl database (http://www.ensembl.org/). All genomic coordinates reported in this study refer to the EquCab 3.0 assembly. A 2.43-Mb candidate region was defined surrounding the lead GWAS variants SNP1 and SNP2 to generate a localized list of annotated genes. This gene set was subsequently analyzed using GeneMANIA^27^ to investigate potential functional relationships and gene-gene interactions. The analysis was executed using default settings, leveraging validated human and mouse biological networks as reference scaffolds to predict functional connectivity.

#### Functional enrichment analysis

To characterize the biological relevance of the identified loci, functional enrichment analysis was performed using the PANTHER v.19.0 Classification System (https://pantherdb.org/).^86^ The 32 genes identified within the candidate regions were assessed for over, or under, representation of Gene Ontology (GO) terms, including biological processes, molecular functions, and biological pathways. Statistical significance was determined using Fisher’s exact test, with Bonferroni correction applied to adjust for multiple testing. The *Equus ferus caballus* and *Mus musculus* genomes were utilized as the reference gene lists for comparative enrichment.
