# Supplementary material for: Machine learning-based gait classification and genome-wide association identify a QTL for gait type in Colombian paso horses
Source: iScience. 2026 Jun 11;29(7):116320. doi: 10.1016/j.isci.2026.116320 (PMC13276308; doi:10.1016/j.isci.2026.116320)
Supplement: Document S1. Figures S1–S5 and Tables S1–S9 [file mmc1.pdf]

iScience, Volume *29*

Supplemental information

Machine learning-based gait classification and genome-wide association identify a QTL for gait type in Colombian paso horses

Miguel Novoa-Bravo, Jennifer R.S. Meadows, Filipe Serra-Bragança, Britt van de Vall, Klas Kullander, Marie Rhodin, and Gabriella Lindgren

Supplemental data titles and legends

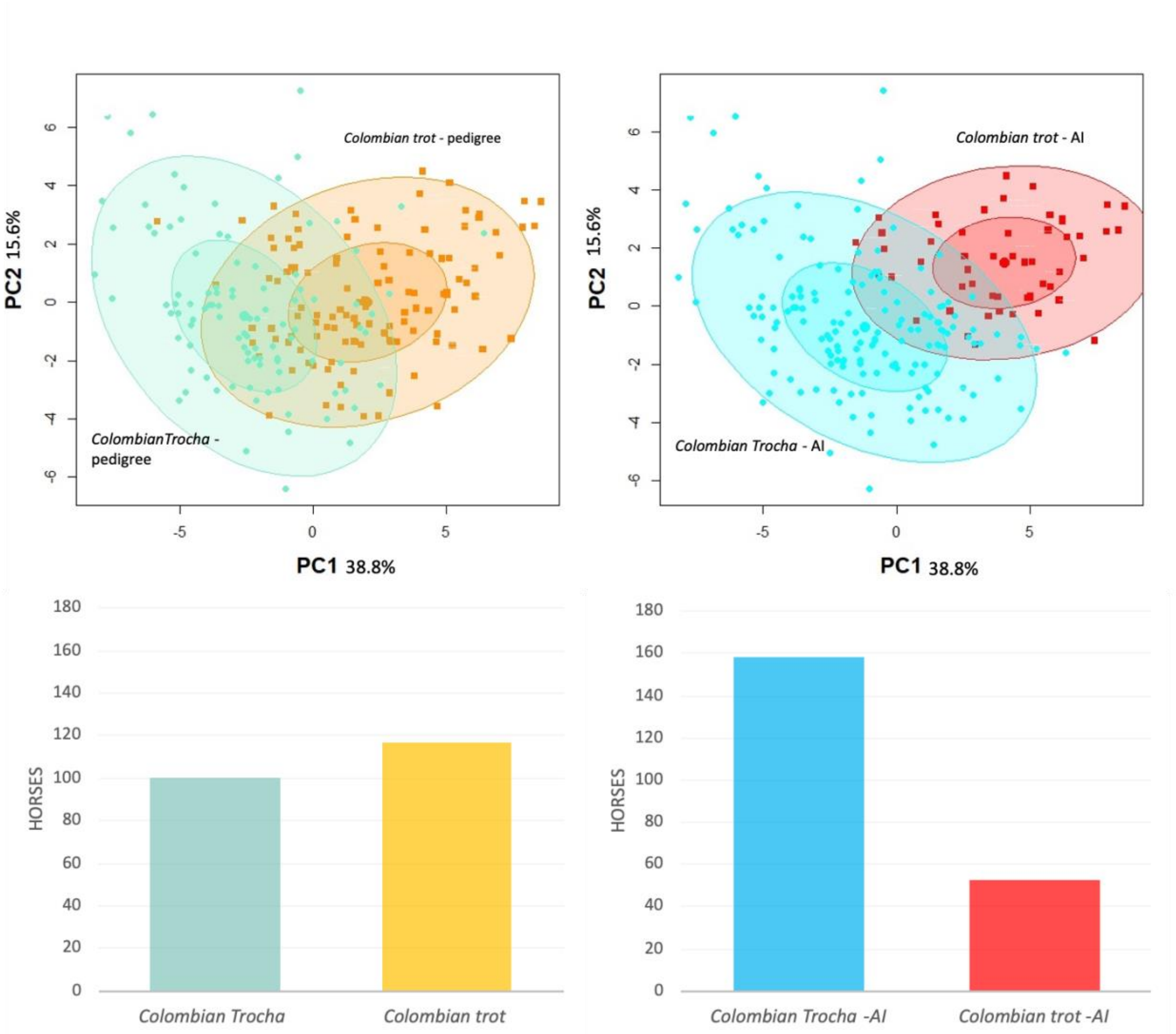

Figure S1. PCA of 34 locomotion variables in 217 Colombian Paso Horses (CPHs), comparing gait classification by pedigree (left) and by AI-LSTM (right), related to Figure 1 and Table 6. Each point represents a horse. Compared with pedigree records, the AI-LSTM algorithm classified 45% fewer horses as Colombian trot and 58% more as Colombian trocha gaited horses. Confidence ellipses show groupings at 90% (lighter) and 95% (darker). Colours: Turquoise=Colombian trocha (pedigree), Orange=Colombian trot (pedigree), Blue=*Colombian trocha* (AI), Red=Colombian trot (AI).

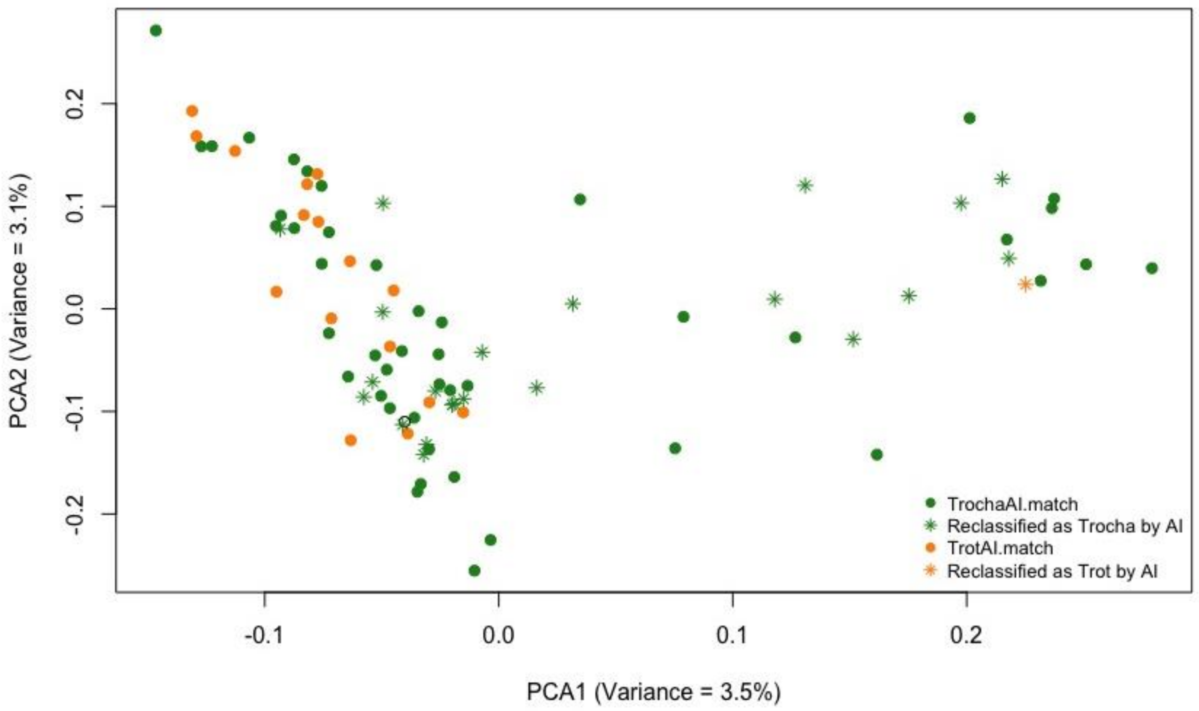

Figure S2. PCA of genomic data from 85 Colombian Paso horses, related to Figure 2. Colour represents the AI-LSTM classification for gait (green, Colombian trocha; orange, Colombian trot) and shape indicates if the pedigree and AI-LSTM classification were concordant (closed circle), or if reclassification was preformed (asterisk). Empty circle was a single horse which was not classified.

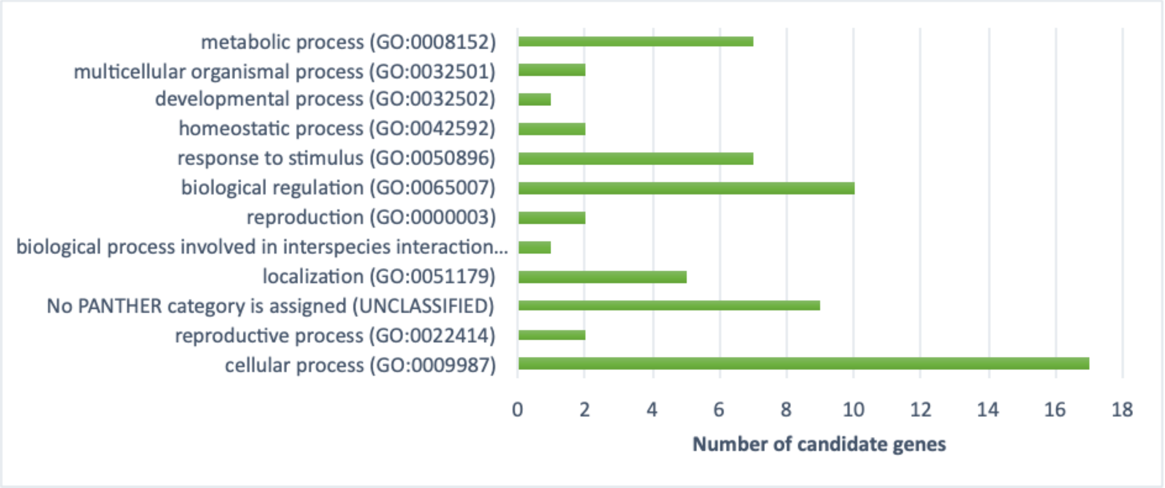

Figure S3. Biological process summary information from the functional classification analysis of the 32 candidate genes for gait class in Colombian Paso Horses in PANTHER[S1]. PANTHER biological process classification: the function of the protein in the context of a larger network of proteins that interact to accomplish a process at the level of the cell or organism.

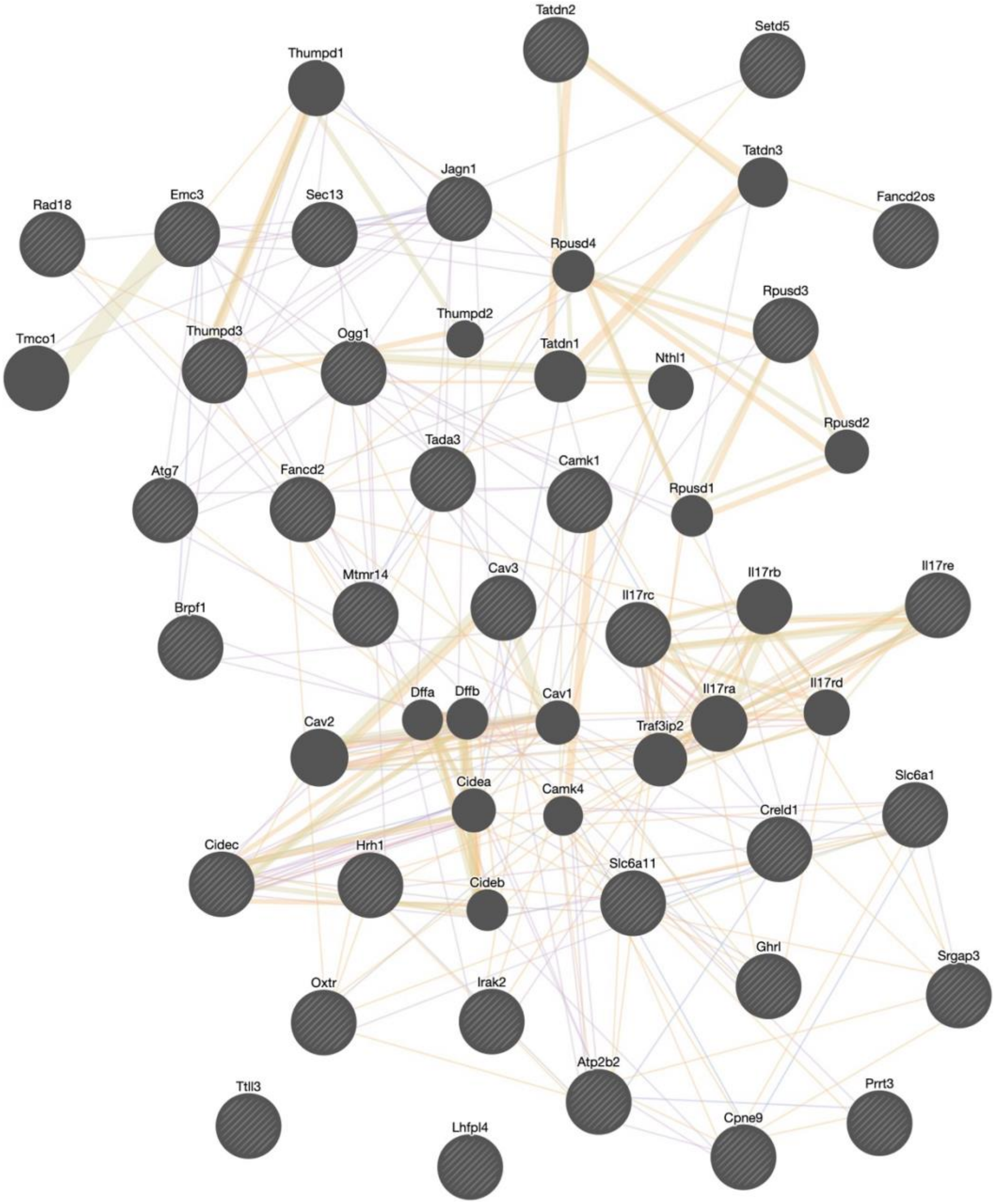

Figure S4. Network of the overlapping genes of the 2.43 Mb region of interest for the QTL in Colombian Paso Horse gaits (Colombian trot vs Colombian trocha), related to Figure 3. Different connecting colours represent different correlations using the mice genome as reference. Genes with stripes were submitted as query terms in searches. Black circles indicate genes associated with query genes.

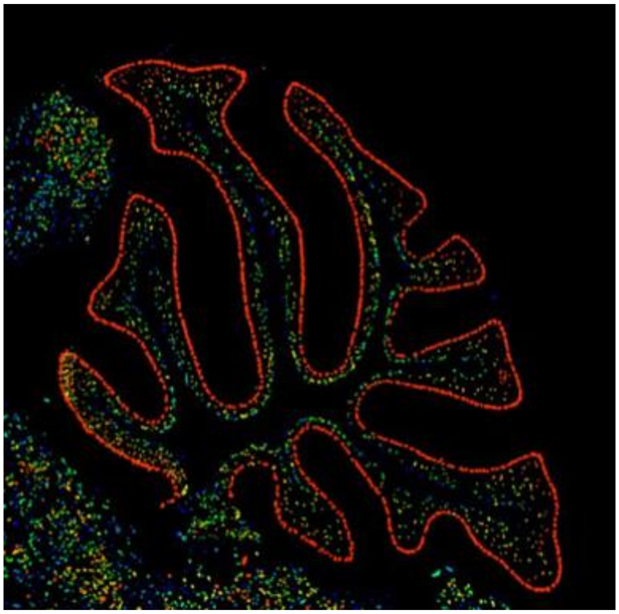

Figure S5. Expression pattern of *ATP2B2* in the mouse cerebellum, red denotes strong expression in Purkinje cells (adapted from the Allen Brain Atlas), related to Figure 3.

Table S1. Description of the features extracted from the IMU sensor data on 225 Colombian Paso Horses related to the locomotion parameters that were measured, related to Figure 1.

**Variable type**	**Variable**	**Unit**	**Description**
Stride timing	Stride duration	s	Duration of one complete stride cycle
	Stance duration (Std)		Period of ground contact (weightbearing) of an individual limb
	Stride frequency (StrFhz)	Hz	Number of repetitions of the stride unit per second
	Duty factor (relative stance duration) (Dt)		Duration of stance phase as a proportion of the total limb cycle duration
Interlimb timing	Diagonal advance placement (DAdvPl)	% of stride duration	Temporal dissociation at hoof contact between diagonal limb pairs
	Lateral advance placement (SAdvPl)		Temporal dissociation at hoof contact between ipsilateral limb pairs
	Minimum number of limbs on the ground		Minimum number of limbs on the ground per stride
	Maximum number of limbs on the ground		Maximum number of limbs on the ground per stride
	Median number of limbs on the ground		Median number of limbs on the ground per stride
	Quadrupedal stance	% of stride duration	Time of simultaneous stance of four limbs
	Tripedal stance		Time of simultaneous stance of tree limbs
	Bipedal stance		Time of simultaneous stance of two limbs
	Single limb stance		Time of simultaneous stance of one limb
	Suspension		Airborne phase of stride where all four limbs are in swing phase and free from weightbearing
	Limb pair overlap LF-RF		Period of synchronous ground contact between LF and RF limbs
	Limb pair overlap LH-RH		Period of synchronous ground contact between LH and RH limbs

	Limb pair overlap LF-LH		Period of synchronous ground contact between LF and LH limbs
	Limb pair overlap RF-RH		Period of synchronous ground contact between RF and RH limbs
	Limb pair overlap LF-RH		Period of synchronous ground contact between LF and RH limbs
	Limb pair overlap RF-LH		Period of synchronous ground contact between RF and LH limbs
Vertical displacement	ROM	cm	Vertical displacement of the sacrum sensor per stride in cm.

L, left; R, Right; F, front; H, hind.

Table S2. Mean and standard deviation of the Inertial Motion Units measured locomotion variables for 225 Colombian Paso horses performing the Colombian trocha or the Colombian trot gait, related to Figure 1. The horses were classified by AI-LSTM algorithm.

Variable	Colombian trot	Colombian trocha
Stride frequency***	2.08 (0.17)	2.43 (0.21)
Stance duration LH***	0.25 (0.02)	0.20 (0.02)
Stance duration LH***	0.25 (0.02)	0.20 (0.02)
Stance duration RF***	0.29 (0.03)	0.23 (0.03)
Stance duration LF***	0.29 (0.03)	0.23 (0.03)
Stride duration***	0.27 (0.02)	0.22 (0.02)
Median limbs ground***	2.20 (0.07)	2.06 (0.09)
Min limbs ground*	1.62 (0.29)	1.02 (0.26)
Max limbs ground*	3.62 (0.27)	3.11 (0.23)
Duty factor lb	52.03 (2.30)	48.98 (2.78)
Duty factor rb	52.26 (2.37)	49.22 (2.64)
Duty factor lf*	60.06 (3.15)	56.65 (4.40)
Duty factor rf*	60.16 (3.22)	56.76 (4.58)
Limb pair overlap LF-RF	50.04 (3.02)	50.17 (5.15)
Limb pair overlap LH-RH	49.37 (3.32)	49.98 (3.87)
Limb pair overlap LF-LH*	-5.61 (3.57)	-7.92 (6.23)
Limb pair overlap RF-RH***	44.20 (4.12)	41.99 (5.06)
Limb pair overlap LF-RH*	52.35 (4.49)	49.79 (4.45)
Limb pair overlap RF-LH	52.19 (3.74)	49.18 (5.08)
Left SAdvPl final*	44.27 (4.28)	34.03 (16.64)
Right SAdvPl final*	43.74 (7.41)	32.42 (19.15)
Left DAdvPl final*	-5.03 (4.63)	-7.95 (5.88)
Right DAdvPl final*	-5.55 (3.58)	-7.77 (6.22)
Quadrupedal stance*	2.501 (1.83)	0.34 (0.54)
Tripedal stance*	16.91 (4.69)	12.36 (6.16)
Bipedal stance*	80.29 (4.65)	81.77 (4.87)
Single limb stance ¢	4.71 (4.18)	15.87 (7.02)
Limb pair overlap LF RF***	19.21 (5.18)	13.55 (6.61)
Limb pair overlap LH RH*	4.63 (2.78)	2.65 (2.43)
Limb pair overlap LF LH***	11.23 (3.48)	7.97 (3.92)

Limb pair overlap RF RH*	11.97 (3.84)	8.75 (4.29)
Limb pair overlap LF RH*	49.83 (2.42)	44.37 (3.20)
Limb pair overlap RF LH*	49.41 (2.36)	44.19 (3.48)
Vertical displacement (rom) *	28.37 (6.17)	30.91 (7.71)

*** p <0.001. T-student
* p <0.001. Nonparametric exact two-sample Kolmogorov-Smirnov test
L, left; R, Right; F, front; H, hind.

Table S3. Count of *DMRT3* genotypes in 217 Colombian Paso Horses classified as performing the Colombian trocha or Colombian trot *gait* using AI-LSTM, related to Figure 2. *NT: not typed.

Gait	*DMRT3*-CA	*DMRT3*-CC	NT	Total
Colombian trot	7	46	-	53
Colombian trocha	13	143	2	158
Misclassified	1	5	-	6

Table S4. Genome-wide association results for the lead SNP associated with gait type (Binary AI-LSTM classification: Colombian *trocha*, n = 67; Colombian *trot*, n = 17) obtained using different genomic models, related to Table 1. The Polygenic mixed model and Eigen-strat 3PCA analyses surpassed the Bonferroni-corrected significance threshold, whereas the other models yielded subthreshold associations.

Genomic Model	Lead SNP	p
Polygenic mixed model	rs1147402472	1.95×10^{-8}
GRAMMAR - gamma	rs1147402472	1.40×10^{-7}
Eigen-strat 3PCA	rs1147402472	1.60×10^{-8}
Genetic clusters	rs1147402472	2.14×10^{-7}
mlma-loco	rs1147402472	4.97×10^{-7}
SAIGE	rs1147402472	3.19×10^{-7}

Table S5. Genes within the ECA16 QTL interval and their potential function in the brain or at a single cell level, related to Table 3. Eleven known genes to be expressed in brain are highlighted in yellow.

Gene	ID	chr:bp	Human Protein Atlas - Brain expression cluster (RNA)	Human Protein Atlas - Single cell type specificity
**Autophagy related 7 – *ATG7***	ENSECAG00000022756	16:7011649-7346033	Non-specific - Mixed function (mainly)	Oocytes
**Histamine receptor H1 – HRH1**	ENSECAG00000004544	16:7283054-7364165	Neurons - Mixed function (mainly)	Secretory cells, Granulosa cells, Glandular and luminal cells, Excitatory neurons, Astrocytes

**Solute carrier family 6 member 1 – SLC6A1**	ENSECAG00000000699	16:7451060-7492629	NA	NA
**Solute carrier family 6 member 11 – SLC6A11**	ENSECAG00000004220	16:7545121-7665662	NA	NA
**ATPase plasma membrane Ca2+ transporting 2 - *ATP2B2***	ENSECAG00000023528	16:7772331-8123695	Neurons & Synapses - Synaptic function (mainly)	Excitatory neurons, Inhibitory neurons, Astrocytes, Horizontal cells, Bipolar cells, Oligodendrocyte precursor cells
**SEC13 homolog, nuclear pore and COPII coat complex component - *SEC13***	ENSECAG00000020459	16:8127669-8156429	Non-specific - Metabolism (mainly)	Low cell type specificity
**Ghrelin and obestatin prepropeptide - *GHRL***	ENSECAG00000025073	16:8166080-8172131	Non-specific - Mixed function (mainly)	Gastric mucus-secreting cells
**TatD DNase domain containing 2 - *TATDN2***	ENSECAG00000010589	16:8176218-8204074	NA	NA
**Interleukin 1 receptor associated kinase 2 - *IRAK2***	ENSECAG00000012960	16:8207107-8284790	Oligodendrocytes - Mixed function (mainly)	Langerhans cells, Schwann cells, Oligodendrocytes, Monocytes, Macrophages
**FANCD2 opposite strand - *FANCD2OS***	ENSECAG00000057625	16:8290006-8313619	Non-specific - Transcription (mainly)	Late spermatids, Early spermatids
**FA complementation group D2 - *FANCD2***	ENSECAG00000018519	16:8291653-8359896	Non-specific - Immune response (mainly)	Spermatocytes, Oocytes, Spermatogonia, Erythroid cells
**ER membrane protein complex subunit 3 - *EMC3***	ENSECAG00000024410	16:8359342-8381594	Low tissue specificity	Early spermatids
**Proline rich transmembrane protein 3 - *PRRT3***	ENSECAG00000014610	16:8385829-8396042	Neurons - Mixed function (mainly)	Rod photoreceptor cells, Horizontal cells
**Protein disulfide-isomerase - *CRELD1***	ENSECAG00000014826	16:8393339-8401391	Neurons - Synaptic function (mainly)	Astrocytes, Excitatory neurons, Inhibitory neurons, Oligodendrocytes
**Interleukin 17 receptor C - *IL17RC***	ENSECAG00000009634	16:8401734-8413462	Non-specific - Mixed function (mainly)	Proximal enterocytes
**Jagunal homolog 1 - *JAGN1***	ENSECAG00000016539	16:8430802-8433900	Non-specific - Metabolism (mainly)	Low cell type specificity

**Cell death inducing DFFA like effector c - *CIDEC***	ENSECAG00000033890	16:8439700-8454855	Not detected - no cluster assigned	Proximal enterocytes
**RNA pseudouridine synthase *D3* - *RPUSD3***	ENSECAG00000019988	16:8453785-8471406	Sub-cortical - Mixed function (mainly)	Early spermatids
**Actin-related protein 2/3 complex subunit 4 - *TTLL3***	ENSECAG00000003474	16:8465465-8484263	Brainstem - Mixed function (mainly)	Low cell type specificity
**ADA3 homolog - *TADA3***	ENSECAG00000018186	16:8497607-8507370	Astrocytes - Mixed function (mainly)	Low cell type specificity
**Calcium/calmodulin dependent protein kinase I - *CAMK1***	ENSECAG00000023628	16:8510815-8527953	Neurons - Mixed function (mainly)	Monocytes, Hofbauer cells
**8-oxoguanine DNA glycosylase - *OGG1***	ENSECAG00000010671	16:8526290-8532932	Non-specific - Transcription (mainly)	Proximal tubular cells, Proximal enterocytes
**Bromodomain and PHD finger containing 1 - *BRPF1***	ENSECAG00000021020	16:8534391-8550130	Non-specific - Transcription (mainly)	Non-specific - Basic cellular processes (mainly)
**Copine family member 9 - *CPNE9***	ENSECAG00000007471	16:8552350-8571912	Hindbrain - Mixed function (mainly)	Late spermatids, Excitatory neurons
**Myotubularin related protein 14 - *MTMR14***	ENSECAG00000020625	16:8572943-8618669	Choroid plexus - Mitochondria (mainly)	Low cell type specificity
**LHFPL tetraspan subfamily member 4 - *LHFPL4***	ENSECAG00000038489	16:8693050-8747375	Neurons - Mixed function (mainly)	Inhibitory neurons, Excitatory neurons, Bipolar cells, Oligodendrocyte precursor cells, Horizontal cells, Rod photoreceptor cells
**SET domain containing 5 - *SETD5***	ENSECAG00000016843	16:8744458-8787229	Non-specific - Transcription (mainly)	Oligodendrocyte precursor cells
**THUMP domain containing 3 - *THUMPD3***	ENSECAG00000002016	16:8790972-8854352	Non-specific - Nucleic acid binding (mainly)	Early spermatids, Late spermatids, Spermatocytes
**SLIT-ROBO Rho GTPase activating protein 3 - *SRGAP3***	ENSECAG00000010118	16:8927898-9169815	Non-specific - Transcription (mainly)	Oligodendrocyte precursor cells
**RING-type E3 ubiquitin transferase - *RAD18***	ENSECAG00000010746	16:9187114-9438340	Non-specific - Mixed function (mainly)	Oocytes
**Oxytocin receptor - *OXTR***	ENSECAG00000017844	16:9437490-9456192	Monoamines - Neurotransmitter signalling (mainly)	Breast myoepithelial cells
**Caveolin 3 - *CAV3***	ENSECAG00000020701	16:9461943-9473116	Not detected - no cluster assigned	Skeletal myocytes, Cardiomyocytes

Table S6. Linkage disequilibrium (LD, r^2) between the two lead SNPs (shown in bold) and additional variants exceeding the suggestive significance threshold, ordered by genomic position along ECA16, related to Table 4. SNP positions are in base pairs; rs, indicates the dbSNP reference identifier.

SNP	Position	*p*	r^2 to SNP1	Functional location
rs396587640	16:7196846	3.9×10^{-6}	0.330	Intronic, (*ATG7)*
**rs1136628503**	**16:7820050**	$\mathbf{7.2\times10^{-8}}$	**0.609**	**SNP2, intronic, (*ATP2B2)***
rs1141932866	16:8206836	3.0×10^{-7}	0.639	Upstream gene variant, (*IRAK2)*
rs394798521	16:8229667	3.5×10^{-7}	0.361	Synonymous variant, (*IRAK2)*
rs1145581384	16:8234676	3.0×10^{-7}	0.639	Intronic, (*IRAK2)*
**rs1147402472**	**16:8860175**	$\mathbf{9.3\times10^{-9}}$	**-**	**SNP1, intergenic**
rs396793867	16:9020017	1.0×10^{-5}	0.794	Intronic, (*SRGAP3)*
rs1151964115	16:9024349	3.7×10^{-7}	0.609	Intronic, (*SRGAP3)*
rs1140383912	16:9624512	4.0×10^{-5}	0.482	Intronic, (*LMCD1)*

Table S7. Haplotype counting within the QTL region associated with gait type as defined by the AI-LSTM classifier (Colombian trot vs. Colombian trocha), related to Table 4.

Haplotype	Sequence[a]	Colombian trot	Colombian trocha	Total
hap1	CCAGCT	1	0	1
hap2	CCAGCC	1	0	1
hap3	CCGATT	2	2	4
hap4	CCGATC	2	0	2
hap5	CCGGCT	0	1	1
hap6	CTGATT	0	0	0
hap7	CTGATC	9	4	13
hap8	TCAATT	0	1	1
hap9	TCAGCT	17	120	137
hap10	TCAGCC	0	3	3
hap11	TCGGCT	0	3	3
hap12	TTAATT	1	0	1
hap13	TTAGCT	1	0	1

[a] Haplotypes are based on six SNPs ordered as follows: rs1147402472 (SNP1), rs1136628503 (SNP2), rs396793867, rs1141932866, rs1145581384, and rs1151964115.

Table S8. Number of horses and frequencies (in brackets) of the associated variants for the 4 additional SNPs[b] in linkage disequilibrium ($r^2 \geq 6$) with the lead SNP1 for gait type QTL in Colombian Paso horses, across 55 horse breeds classified as gaited or not gaited, related to Table 5.

Breed	SNP3-G	SNP4-A	SNP5-T	SNP6-C	Gait
**Gaited horses**					
Akhal-Teke	4 (0.00)	4 (0.38)	4 (0.25)	4 (0.00)	Glide[a]

Aegidienberger	2 (0.00)	2 (0.50)	2 (0.00)	2 (0.00)	Tolt, pace
American Curly	2 (0.25)	2 (0.00)	2 (0.00)	2 (0.00)	Foxtrot
American Miniature Horse	5 (0.30)	5 (0.00)	4 (0.13)	5 (0.00)	Pace
American Saddlebred	2 (0.00)	2 (0.00)	2 (0.00)	2 (0.00)	Harness, pace
American Standardbred	41 (0.05)	41 (0.05)	41 (0.09)	41 (0.07)	Harness, pace
Brazilian Mangalarga Marchador	1 (0.00)	1 (0.00)	-	1 (0.00)	Marcha
Chinese Mongolian Horse	100 (0.02)	100 (0.08)	100 (0.09)	100 (0.10)	Joroo
French Trotter	10 (0.40)	10 (0.00)	10 (0.00)	10 (0.05)	Harness
Icelandic horse	397 (0.49)	396 (0.02)	397 (0.03)	397 (0.15)	Tolt, pace
Missouri Fox Trotter	1 (0.00)	1 (0.50)	1 (0.00)	1 (0.00)	Foxtrot, pace
Morgan	20 (0.30)	20 (0.05)	20 (0.28)	20 (0.10)	Single-foot
Native Mongolian Chakouyi	1 (0.00)	1 (0.50)	-	1 (0.00)	Pace
Tennessee Walking Horse	4 (0.13)	4 (0.00)	3 (0.00)	4 (0.00)	Running walk
Welsh Pony	22 (0.25)	22 (0.05)	22 (0.32)	22 (0.02)	Pace
Total gaited horses	**612 (0.38)**	**611 (0.04)**	**608 (0.06)**	**612 (0.12)**	
**Non-gaited horses**					
American Paint Horse	4 (0.13)	4 (0.13)	4 (0.00)	4 (0.00)	Not gaited
Arabian Horse	23 (0.15)	23 (0.09)	22 (0.07)	23 (0.00)	Not gaited
Austrian Warmblood	1 (0.00)	1 (0.00)	1 (0.00)	1 (0.00)	Not gaited
Badenwürttem-bergisches Warmblut	1 (0.00)	1 (0.50)	1 (0.00)	1 (0.00)	Not gaited
Bayrisches Warmblut	1 (0.50)	1 (0.00)	1 (0.00)	1 (0.00)	Not gaited
Belgian Warmblood	2 (0.50)	2 (0.75)	2 (0.50)	2 (0.25)	Not gaited
British Warmblood	5 (0.13)	5 (0.10)	5 (0.00)	5 (0.40)	Not gaited
Clydesdale	19 (0.05)	19 (0.08)	19 (0.13)	19 (0.00)	Not gaited
Connemara	4 (0.50)	4 (0.13)	4 (0.38)	4 (0.00)	Not gaited
Czech Republic Warmblood	1 (0.00)	1 (0.00)	1 (0.00)	1 (0.00)	Not gaited
Danish warmblood	2 (0.00)	2 (0.00)	2 (0.00)	2 (0.00)	Not gaited
Dutch Warmblood	4 (0.13)	4 (0.13)	4 (0.00)	4 (0.00)	Not gaited
Franches-Montagnes	60 (0.32)	60 (0.00)	60 (0.07)	60 (0.28)	Not gaited
French Warmblood	37 (0.11)	37 (0.14)	37 (0.08)	37 (0.07)	Not gaited
German Riding Pony	2 (0.00)	2 (0.25)	2 (0.00)	2 (0.00)	Not gaited
German Warmblood	114 (0.11)	114 (0.14)	114 (0.08)	114 (0.03)	Not gaited
Haflinger	11 (0.41)	11 (0.18)	11 (0.05)	11 (0.09)	Not gaited
Hannoveraner	5 (0.00)	5 (0.10)	5 (0.00)	5 (0.00)	Not gaited
Holsteiner	11 (0.09)	11 (0.09)	11 (0.14)	11 (0.09)	Not gaited
Irish Warmblood	2 (0.25)	2 (0.00)	2 (0.00)	2 (0.25)	Not gaited
Japanese Thoroughbred Horse	370 (0.04)	370 (0.01)	370 (0.00)	-	Not gaited

Koninklijk Warmbloed Paard	1 (0.50)	1 (0.50)	1 (0.50)	1 (0.50)	Not gaited
Latvian Warmblood	1 (0.50)	1 (0.50)	1 (0.50)	1 (0.00)	Not gaited
Lipizzaner	4 (0.63)	4 (0.50)	4 (0.25)	4 (0.63)	Not gaited
Noriker	1 (0.00)	1 (0.00)	1 (0.00)	1 (0.00)	Not gaited
Norwegian Fjord	1 (0.00)	1 (0.00)	1 (0.00)	1 (0.00)	Not gaited
Oldenburger	5 (0.00)	5 (0.00)	5 (0.10)	5 (0.00)	Not gaited
Percheron	4 (0.25)	4 (0.00)	3 (0.00)	4 (0.25)	Not gaited
Polish Warmblood	2 (0.00)	2 (0.00)	2 (0.00)	2 (0.00)	Not gaited
Pure Spanish breed	1 (0.50)	1 (0.00)	1 (0.00)	1 (0.50)	Not gaited
Quarter Horse	55 (0.17)	55 (0.08)	55 (0.05)	55 (0.05)	Not gaited
Russian Warmblood	1 (0.00)	1 (0.00)	1 (0.00)	1 (0.00)	Not gaited
Shetland Pony	23 (0.20)	23 (0.00)	23 (0.09)	23 (0.07)	Not gaited
Sorraia	1 (1.00)	1 (0.50)	1 (0.00)	1 (1.00)	Not gaited
Swedish Warmblood	2 (0.00)	2 (0.00)	2 (0.00)	2 (0.00)	Not gaited
Swiss Warmblood	214 (0.17)	214 (0.13)	214 (0.10)	214 (0.04)	Not gaited
Thoroughbred	29 (0.02)	29 (0.00)	29 (0.10)	29 (0.02)	Not gaited
Trakehner	5 (0.00)	5 (0.00)	5 (0.00)	5 (0.00)	Not gaited
Westphalian	4 (0.25)	4 (0.25)	4 (0.13)	4 (0.25)	Not gaited
Yakut	9 (0.28)	9 (0.00)	9 (0.06)	9 (0.22)	Not gaited
Total non-gaited horses	**1042 (0.12)**	**1042 (0.07)**	**1040 (0.06)**	**672 (0.08)**	

[a] Classified as gaited horse breed according to previous work [S2].
[b] SNP3 (rs396793867), SNP4 (rs1141932866), SNP5 (rs1145581384), and SNP6 (rs1151964115).

Table S9. Haplotypes frequencies in 10 horse breeds using the lead SNP1, SNP2 and the 4 additional SNPs in linkage disequilibrium ($r^2 \geq 0.6$) for the gait type QTL in Colombian Paso Horses, related to Table 5. Data from the European Variation Archive. The haplotype associated with Colombian trot gait (CTGACT) is absent in these breeds and the haplotype associated with Colombian trocha gait (TCAGCT) is in bold.

	**Gaited Breeds[a]**			**Non-gaited Breeds[b]**						
**Haplotype[c]**	**ICE**	**CMH**	**AS**	**JPN**	**FM**	**QH**	**FW**	**GW**	**SW**	**THO**
CCAATT	0	0	0	0	0	0	0.04	0.01	0.01	0
CCAGCC	0	0	0.05	0	0.02	0	0	0	0	0
CCAGCT	0.02	0.07	0.1	0.05	0.11	0.1	0.06	0.02	0.09	0.02
CCGACT	0	0	0.04	0	0	0.01	0	0	0	0
CCGATT	0	0.01	0	0	0	0	0	0.03	0.07	0
CCGGCC	0.03	0.01	0	0	0.1	0.01	0	0.01	0	0
CCGGCT	0.16	0.02	0	0	0.05	0	0	0	0	0
CTAGCT	0.04	0	0	0	0	0	0.01	0	0	0
TCAACC	0	0	0	0	0	0.02	0	0	0	0
TCAACT	0	0	0	0	0	0	0.05	0	0.02	0
TCAATT	0.01	0.04	0	0	0	0	0	0.02	0	0

TCAGCC	0.02	0.01	0.03	0	0.14	0.01	0	0.01	0.01	0.02
**TCAGCT**	0.35	0.52	0.67	0.61	0.38	0.54	0.69	0.59	0.53	0.59
TCAGTT	0.02	0.01	0.05	0	0.03	0.01	0	0	0	0.07
TCGGCC	0.05	0.06	0	0	0	0	0.01	0	0.02	0
TCGGCT	0.19	0.09	0	0.03	0.13	0.06	0.05	0.03	0.06	0
TTAACT	0	0.01	0	0	0	0	0	0.04	0.01	0
TTAGCT	0.04	0.07	0.03	0.29	0	0.13	0.03	0.18	0.14	0.25
TTAGTT	0	0	0.03	0	0	0.01	0	0	0	0.04
TTGGCC	0.03	0	0	0	0	0	0.01	0	0	0

[a] Gaited horse breeds [S2] : Icelandic (ICE, n= 397), Chinese Mongolian Horse (GHM, n= 100), American Standardbred (AS, n= 41).
[b] Thoroughbred-Japan (JP, n= 370), Franches-Montagnes (FM, n= 60), Quarter Horse (QH, n= 55), French Warmblood (FW, n= 37), Swiss Warmblood (SW, n= 214), Thoroughbred (THO, n= 28), and German Warmblood (GW, n= 114).
[c]The sequence order of the SNPs is: rs1147402472 (SNP1), rs1136628503 (SNP2), rs396793867 (SNP3), rs1141932866 (SNP4), rs1145581384 (SNP5), and rs1151964115 (SNP6).

Supplemental references

S1. Mi, H., Ebert, D., Muruganujan, A., Mills, C., Albou, L.P., Mushayamaha, T., and Thomas, P.D. (2021). PANTHER version 16: a revised family classification, tree-based classification tool, enhancer regions and extensive API. Nucleic Acids Res. 49, D394–D403. https://doi.org/10.1093/NAR/GKAA1106.

S2. Nicodemus, M.C., and Clayton, H.M. (2003). Temporal variables of four-beat, stepping gaits of gaited horses. Appl. Anim. Behav. Sci. 80, 133–142. https://doi.org/10.1016/S0168-1591(02)00219-8.
